# Solid-state NMR of membrane peptides and proteins in the lipid cubic phase

**DOI:** 10.1016/j.bpj.2025.03.012

**Published:** 2025-03-20

**Authors:** Kiefer O. Ramberg, Coilin Boland, Hamed Kooshapur, Olivier Soubias, Maciej Wiktor, Chia-Ying Huang, Jonathan Bailey, Klaus Gawrisch, Martin Caffrey

**Affiliations:** 1 Membrane Structural & Functional Biology Group, School of Medicine and School of Biochemistry & Immunology, Trinity College Dublin, D02 R590 Dublin, Ireland; 2 Laboratory of Structural Biophysics, Biochemistry and Biophysics Center, National Heart, Lung, and Blood Institute, National Institutes of Health, Bethesda, Maryland; 3 Macromolecular NMR Section, Center for Structural Biology, Center for Cancer Research, National Cancer Institute, National Institutes of Health, Frederick, Maryland; 4 Laboratory of Biochemistry, Faculty of Biotechnology, University of Wrocław, Wrocław, Poland; 5 Swiss Light Source, Center for Photon Science, Paul Scherrer Institute, Forschungsstrasse 111, 5232 Villigen PSI, Switzerland; 6 Laboratory of Membrane Biochemistry and Biophysics, National Institute on Alcohol Abuse and Alcoholism, National Institutes of Health, Bethesda, Maryland

## Abstract

Solid-state nuclear magnetic resonance (ssNMR) is a powerful technique for studying membrane protein structure and dynamics. Ideally, measurements are performed with the protein in a lipid bilayer. However, homogenous reconstitution of functional protein into intact bilayers at sufficiently high concentrations is often difficult to achieve. In this work, we investigate the suitability of the lipid cubic phase (LCP), which incorporates a lipid bilayer, as an alternative medium for ssNMR of integral membrane peptides and proteins. The cubic mesophase has long been used to generate membrane protein crystals for use in X-ray crystallographic structure determination by the so-called in meso method and for protein functional and biophysical characterization. Preparing and handling protein-laden LCP is straightforward. LCP may therefore provide a valuable alternative to native membranes and other membrane mimetics for ssNMR. We tested this idea by conducting standard magic-angle spinning ssNMR experiments on LCP into which gramicidin, a ∼4-kDa transmembrane peptide, or bacterial lipoprotein signal peptidase II (LspA), a ∼20-kDa integral membrane enzyme, had been reconstituted. We report one- and two-dimensional ssNMR spectra for both gramicidin and LspA and the parameters for optimizing spectral quality. The high protein-carrying capacity of the cubic phase facilitated ^13^C ssNMR at natural abundance. Lowering temperature and raising magic-angle spinning frequency enabled significant improvements in spectral quality. One-dimensional ^13^C and ^15^N spectra were collected for LspA. Two-dimensional ssNMR experiments provided information on LspA dynamics and its interaction with the water and lipid components of the cubic phase. Solution NMR measurements carried out in parallel yielded information on the effect of the antibiotic, globomycin, on LspA structure and dynamics.

## Significance

Solid-state NMR (ssNMR) is an atomic-level method for determining the structure and dynamics of membrane proteins that complements more static structural information provided by crystallography, single particle cryoelectron microscopy, and prediction methods. Since preparation of protein-laden membranes for ssNMR can be challenging, more convenient membrane mimetic systems are needed. Reconstituting membrane proteins into the lipid cubic phase (LCP) is straightforward, enabling the crystallization of many “sensitive” drug targets. Here, we demonstrate that the protein-laden LCP can be used to collect NMR spectra of membrane proteins and peptides. This work should aid implementation of the LCP as a convenient membrane mimetic for ssNMR of membrane proteins and peptides in the drug-development pathway.

## Introduction

Membrane proteins perform a myriad of critical cellular functions and are high-profile drug targets (1,2,3). Until recently, structure-based design of drugs targeting membrane proteins has largely relied on macromolecular X-ray crystallography (MX) (4,5,6,7,8). However, MX requires the challenging step of growing crystals and may suffer from what has been referred to as the “tyranny of the lattice,” where unnatural conformations can be encountered (9). Solid-state NMR (ssNMR) affords the opportunity to study integral membrane proteins in native-like membrane environments that are difficult to mimic in more traditional MX studies where targets are reconstituted in detergent micelles. The information forthcoming from an ssNMR experiment includes not only local structure at atomic resolution but also dynamics for a more complete insight into physiological function (3,10,11,12,13,14,15,16,17,18,19,20,21,22).

**Figure 1:**
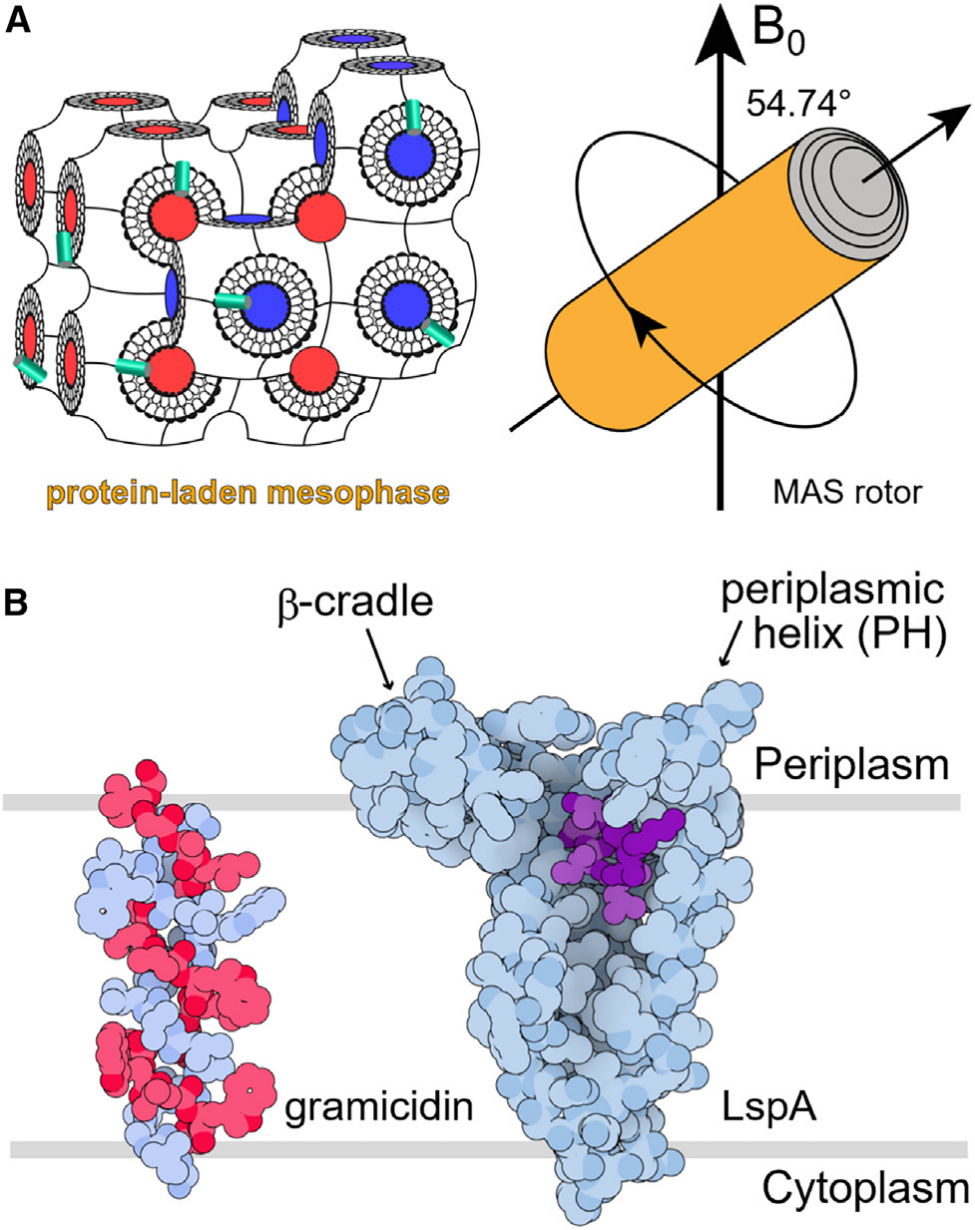
Materials and methods used to perform ssNMR measurements on membrane peptides and proteins in the lipid cubic phase. (*A*) Schematized view of a membrane protein-laden bicontinuous LCP (*left*) packed into a ssNMR MAS rotor (*right*). Spinning is around the long axis of the rotor which is tilted at the magic angle of 54.74° with respect to the magnetic field (B_0_) direction (*vertical arrow*). The mesophase consists of a highly curved, continuous lipid bilayer, both sides of which are water coated. The two water channels (*blue and red*) interpenetrate but never contact one another because they are separated by a lipid bilayer. Proteins embedded in the LCP bilayer are represented as cyan-colored cylinders. (*B*) In meso crystal structures of gramicidin (PDB: 2XDC) and globomycin-bound LspA (PDB: 5DIR). Protein and peptide chains (*blue and red*) and globomycin (*purple*) are in space-filling representation. Approximate membrane boundaries are indicated by horizontal lines. For a Figure360 author presentation of this figure, see https://doi.org/10.1016/j.bpj.2025.03.012.

Although mixed lipid bilayers are the most biologically relevant environments in which to study membrane proteins (10,11,12,13,14), the preparation of homogenous samples of protein-laden oriented bilayers for ssNMR is challenging due to the technical nature of aligning hydrated membranes mechanically between stacks of glass plates. Easier-to-handle mimetics employed successfully to date include nanodisks (15), macrodisks (16), bicelles (17,18), liquid crystals (19), and liposomes (20,21,22). Another potentially useful membrane mimetic for ssNMR of membrane proteins is the lipid cubic phase (LCP). The LCP is a liquid crystalline material that, at its simplest, consists of approximately equal parts lipid and water. The lipid, typically a monoacylglycerol (MAG) such as monoolein, adopts the form of a continuous bilayer that is highly curved and multiply branched. The bilayer is hydrated on either of its polar surfaces giving rise to two continuous, interpenetrating but noncontacting aqueous channels (Fig. 1 *A*). The LCP has been used to grow crystals of membrane proteins for structure determination by MX with over 1000 so-called in meso structures deposited in the Protein Data Bank (PDB) to date (4). For crystallogenesis, the protein is initially reconstituted into the bilayer of the cubic phase. The protein-laden mesophase is then incubated with precipitant solutions of differing compositions under controlled conditions of temperature to facilitate crystal growth (23). Interestingly, structured lipids commonly appear in the solved crystal structure where they often adopt a bilayer arrangement around the protein mimicking the native membrane from which the protein originated (5,6,7). The LCP has also been employed as a membrane mimetic in which to functionally and biophysically characterize membrane proteins (24,25) and has been used as a system in which to refold a denatured membrane protein into its biologically active and crystallizable form (26).

Although the cubic mesophase has been extensively studied by NMR (27,28,29), its potential for use in characterizing membrane proteins by ssNMR has not been explored in detail. Solution NMR has been used to investigate small transmembrane and membrane peripheral peptides in the LCP (30). However, only signals from the N- and C-terminal regions of the peptide could be observed, whereas the bilayer-embedded residues went undetected. In parallel, solution NMR measurements with a peripheral or membrane-associating peptide in the same LCP sample setting yielded signals for most peptide residues, indicating that confinement in the aqueous channels of the mesophase did not drastically restrict peptide tumbling (30). In a separate study, well-resolved solution NMR spectra were obtained for the ∼6 kDa soluble protein, GB1, in the LCP with sequestration in the mesophase aqueous channels apparently having negligible effect on the tertiary structure of the protein (31). ssNMR experiments have been carried out on membrane-protein-containing LCP samples to probe the sterol-binding sites of the *β*_2_-adrenergic G protein-coupled receptor (32). In this case, the reporter was ^13^C-labeled cholesterol, whereas the receptor was unlabeled and went undetected. In the current work, we present ssNMR spectra of a membrane peptide and a membrane protein incorporated into the LCP.

Gramicidin is a 15-residue antimicrobial peptide of form: formyl-NH-L-Val(Ile)^1^-Gly^2^-L-Ala^3^-D-Leu^4^-L-Ala^5^-D-Val^6^-L-Val^7^-D-Val^8^-L-Trp^9^-D-Leu^10^-L-Trp(Phe,Tyr)^11^-D-Leu^12^-L-Trp^13^-D-Leu^14^-L-Trp^15^-NHCH_2_CH_2_OH (The linear gramicidin used in this study is a mixture of gramicidins A, B, and C, which differ in the identity of the first and 11^th^ amino acids as shown in brackets in the sequence (32).). It functions by creating pores in membranes that trigger the collapse of vital transmembrane ion gradients. Gramicidin has long been used as a model for integral membrane proteins with a small footprint in the membrane plane. These include single helical transmembrane proteins, also known as single spanners, for which structural knowledge is in short supply but badly needed in light of their enormous physiological relevance and potential as drug targets (33,34,35,36). Gramicidin has been characterized in a variety of conformational states by NMR and by MX. NMR data for the peptide in lipid bilayers (14,37) and detergent micelles (38,39) are consistent with a stacked assembly of right-handed helical dimers widely accepted as the functional pore-forming form of the peptide. Spectroscopic measurements of gramicidin reconstituted into the cubic phase also suggested a helical dimer assembly (40). However, limited in meso MX studies have revealed only the antiparallel helical homodimer arrangement (Fig. 1 *B*) (41,42). The absence of alternate gramicidin assemblies, including the right-handed helical dimer form, may be a consequence of the presence of polyethylene glycol (PEG) in the precipitant solution, which can cause the gramicidin-laden LCPs to undergo a transition to the sponge phase (42). It has been suggested that, although gramicidin may exist in different assembly states upon reconstitution into the LCP, the PEG-induced phase transition and/or interactions between PEG and gramicidin, as observed in the solved structures, may stabilize the antiparallel dimer assembly in meso.

The larger, 169-residue LspA is a key enzyme in the lipoprotein processing pathway of *Pseudomonas aeruginosa* (5). It has orthologs in many other human pathogens, including methicillin-resistant *Staphylococcus aureus* (6). LspA functions to release the signal peptide from prolipoproteins and is inhibited by the natural macrocyclic antibiotics, globomycin, and myxovirescin (6). It is a promising target for the development of drugs that combat antimicrobial resistance. In meso crystal structures of LspA in complex with globomycin and myxovirescin reveal that the enzyme consists of four transmembrane helices with periplasmic extensions that include an amphiphilic *β* sheet region, referred to as the *β* cradle, and a flexible loop with a short periplasmic helix (Fig. 1 *B*) (5,6). Solution NMR (43) and molecular dynamics simulations (44) studies have led to proposals for different conformational states adopted by the protein with and without bound antibiotic and have highlighted significant structural rearrangements in the *β* cradle and periplasmic helix regions upon inhibitor binding.

Herein, we demonstrate ssNMR of gramicidin and LspA reconstituted in the LCP. Using standard ssNMR techniques with magic-angle spinning (MAS), spectra for gramicidin and LspA were obtained. The effects of temperature and MAS frequency on spectral signal intensity and resolution were investigated. Data collection temperature and MAS frequency values providing best spectra are reported. Performing an in meso ssNMR experiment involves preparing isotopically labeled protein-laden mesophase as per the established coupled syringe-mixing method used to set up in meso crystallization trials (23). LCP in a Hamilton syringe is loaded into an MAS rotor and ^1^H-, ^13^C-, and ^15^N-detected spectra are collected using established ssNMR experiments. In terms of signal intensity and resolution, the recorded spectra are comparable to previously reported membrane protein ssNMR data (3,10,11,12,13,14,15,16,17,18,19,20,21,22) and showcase the LCP as an effective membrane mimetic for ssNMR of membrane-protein targets. Given the high intrinsic curvature of the membrane in the cubic phase and the fact that curvature can be adjusted over a wide range, the LCP may prove useful as a medium in which to investigate physiological processes, such as membrane fusion and fission, by ssNMR.

## Materials and methods

### Gramicidin-LCP sample preparation

An optically clear and nonbirefringent sample of gramicidin-containing LCP was prepared as described previously (40,41,42). The procedure is illustrated in Fig. S1. Monoolein (9.9 MAG; Nu-Chek, Elysian, MN, USA; lot M239-D9-Y) and linear gramicidin from *Bacillus brevis*^1^ (Sigma-Aldrich, Saint Louis, MO, USA; cat. no. G5002; lot 089K1024) as dry solids were combined in 2,2,2-trifluoroethanol (TFE) at a molar ratio of 20:1. The suspension was shaken by hand at room temperature (RT; 20°C–22°C) for ∼3 min until the sample became optically clear. TFE was evaporated under a stream of nitrogen gas and the sample was dried fully under high vacuum (10 mbar; Büchi Vac V500, Büchi, Flawil, Switzerland) at 20°C for approximately 24 h. The dry gramicidin/monoolein mixture was heated at 42°C until molten and was then transferred into a 100 *μ*L gas-tight Hamilton syringe (Hamilton Company, Reno, NV, USA). All subsequent steps were performed at RT. 25 mM sodium potassium phosphate buffer pH 5.6 was transferred to a second 100 *μ*L gas-tight Hamilton syringe at a 3:2 weight ratio of dry monoolein/gramicidin to buffer, as described previously (23). Each syringe was fitted with a Teflon ferrule before being connected using a metal coupling device constructed in house by combining two removable needles (gauge 22; Hamilton Company, Reno, NV, USA; cat. no. 7770-020) and two removable needle nuts (Hamilton Company, Reno, NV, USA; cat. no. 30902) as described (45). The molten monoolein/gramicidin and buffer were combined by passing the contents of one syringe into the other through the coupler (Fig. S1). The combined mixture was passed back and forth into each syringe via the coupler until it became optically clear. Successful formation of LCP was evaluated by inspecting the syringe contents between crossed polarizing filters. The characteristic viscosity along with the absence of birefringence confirmed the successful generation of the LCP, which is optically isotropic (23). The coupler was kept attached to the syringe in which the sample was being stored and was used to transfer the gramicidin-LCP sample directly into a 4-mm Bruker ssNMR MAS rotor.

### *In vivo* production and purification of ^15^N-labeled LspA

The sequence for LspA from *P. aeruginosa* PAO1 (UniProt: Q9HVM5) modified N-terminally with an MGSS sequence followed by a hexa-histidine tag, a spacer sequence (SSG), and the thrombin cleavage site LVPRGSH (Fig. S2 A) was previously cloned into the pET-28a expression vector (kan^r^) and transformed into competent *Escherichia coli* C41 (DE3) cells (5). Freshly transformed cells were spread onto an LB (Luria Broth) agar plate and incubated overnight (16 h) at 37°C. The next day, a single colony was used to inoculate 60 mL of LB medium in a 250 mL Erlenmeyer flask and incubated overnight (16 h) at 37°C and 180 rpm. All cultures were supplemented with 50 *μ*g/mL kanamycin. The next day, the overnight culture was used to inoculate six 3 L baffled flasks (10 mL of overnight culture per flask) containing 1 L of M9 medium supplemented with 1 g/L ^15^N ammonium chloride (Cambridge Isotope Laboratories, Tewksbury, MA, USA; cat. no. NLM-467) and kanamycin (50 *μ*g/mL) and incubated at 37°C and at 180 rpm. The M9 medium was made by adding the following to 867 mL of Milli-Q water: 100 mL of 10× M9 salt solution containing Na_2_HPO_4_ (337 mM), KH_2_PO_4_ (222 mM), and NaCl (85.5 mM); 20 mL of D-glucose (20%(w/v)); 1 mL of MgSO_4_ (1 M); 0.3 mL of CaCl_2_ (1 M); 1 mL of biotin (1 mg/mL); 1 mL of thiamine (1 mg/mL); and 10 mL of 100× trace-element solution containing EDTA (13.4 mM), FeCl_3_-6H_2_O (3.1 mM), ZnCl_2_ (0.62 mM), CuCl_2_-2H_2_O (76 *μ*M), CoCl_2_-2H_2_O (42 *μ*M), H_3_BO_3_ (162 *μ*M), and MnCl_2_-4H_2_O (8.1 *μ*M). Cultures were grown at 37°C and 180 rpm to an optical density at 600 nm (OD_600_) of 0.5 at which point the temperature was reduced to 30°C. Upon reaching an OD_600_ of 0.6, LspA expression was induced by the addition of isopropyl *β*-D-1-thiogalactopyranoside (IPTG) to a final concentration of 1 mM. The cultures were grown for a further 20 h at 30°C and 180 rpm. The cells were harvested by centrifugation at 6000 × *g* for 10 min using a Sorvall RC5C+ centrifuge with an F10S-6x500y rotor at 4°C. 1 M stocks of 2-(N-morpholino)ethanesulfonic acid (MES) used to prepare purification buffers were pH-adjusted to 6.15 with NaOH at 4°C. The cell pellet was resuspended in buffer A (50 mM MES-NaOH pH 6.15, 150 mM NaCl, 10%(v/v) glycerol) using 1 mL of buffer per gram of pellet. A single EDTA-free cOmplete Protease Inhibitor Cocktail tablet (Roche, Basel, Switzerland; cat. no. 11836170001) was added per 50 mL of resuspended cells. The cells were lysed by passaging three times through an Emulisflex C5 cell disruptor at 17,000 psi and 4°C. The lysed cells were centrifuged at 100,000 × *g* for 60 min at 4°C using a Beckman Coulter Optima L-100Xultra centrifuge fitted with a Ti70 rotor and the membrane pellet was resuspended in buffer A containing 1%(w/v) Fos-choline-12 (FC-12) and mixed for 1.5 h at 4°C. The unsolubilized material was pelleted by centrifugation at 100,000 × *g* for 60 min at 4°C using a Beckman Coulter Optima L-100X Ultracentrifuge fitted with a Ti70 rotor. The supernatant was incubated with 3 mL of nickel-nitrilotriacetic acid (Ni-NTA) resin (Cube Biotech, Monheim, Germany; cat. no. 74103), pre-incubated in buffer B (50 mM MES pH 6.15, 150 mM NaCl, 10%(v/v) glycerol, 0.14%(w/v) FC-12), for 1 h at 4°C. The resin was washed with 10 column volumes (CV) of buffer B followed by 20 CV of buffer B supplemented with 50 mM imidazole. The bound protein was eluted with buffer B supplemented with 300 mM imidazole. ^15^N-labeled LspA was further purified using a HiLoad 16/60 Superdex 200 gel filtration column equilibrated with buffer C (50 mM MES-NaOH pH 6.15, 150 mM NaCl, 10%(v/v) glycerol, and 0.14%(w/v) FC-12). Protein homogeneity was assessed by analytical SEC using a Superdex 200 10/300 GL column equilibrated with buffer C (Fig. S2 B). The peak fractions were concentrated using a Millipore centrifuge filter with a molecular-weight cutoff of 50 kDa, snap-frozen in liquid nitrogen, and stored at −70°C until required. SEC-purified protein was analyzed by SDS-PAGE and Coomassie staining (Fig. S2 C). Protein concentration was determined spectrophotometrically at 280 nm using an extinction coefficient of 55,460 M^−1^⋅cm^−1^ as determined using ProtParam (46). The protein was not subjected to thrombin cleavage and was used in its hexa-histidine tagged state.

### Cell-free production and purification of ^2^H/^13^C/^15^N-labeled LspA

^13^C/^15^N- and ^2^H/^13^C/^15^N-labeled forms of the LspA construct described above were produced using a continuous exchange *E. coli*-based precipitate cell-free system as described previously (47). The same plasmid used for *in vivo* production of LspA was employed for cell-free expression. Briefly, preparative-scale expression was conducted using 3 mL of reaction mix and 42 mL of feeder mix supplemented with either ^13^C/^15^N- or ^2^H/^13^C/^15^N-labeled amino acid mixtures (Sigma-Aldrich, Saint Louis, MO; cat. nos 767964 and 771031) (48). Expression was carried out in an incubator at 30°C with continuous shaking at 150 rpm for 16 h. Precipitated material containing LspA from the cell-free reaction was harvested from the reaction cassette (Thermo Fisher Scientific, Waltham, MA; cat. no. 66380) using a 5 mL syringe fitted with a 22-gauge needle. The precipitated material was harvested by centrifugation at 12,000 × *g* for 10 min at 4°C using a Sorvall RC5C+ centrifuge fitted with an SS34 rotor. The pelleted material was resuspended in buffer A (described above) and was subjected to the same solubilization procedure as described for *in vivo*-produced ^15^N-labeled LspA. After solubilization, the purification of the solubilized protein was carried out as for *in vivo* production. Homogeneity and purity were monitored by SEC and SDS-PAGE, respectively (Figs. S3 and S4). Higher-molecular-weight bands observed by SDS-PAGE were identified as LspA by transferring SDS-PAGE gel content to nitrocellulose membrane using an iBlot transfer system (Thermo Fisher Scientific, Waltham, MA; cat. no. 10010178). The membrane was blocked with 1.5%(w/v) BSA in Tris-buffered saline for 1 h before adding anti-polyhistidine-peroxidase antibody (Sigma-Aldrich, Saint Louis, MO, USA; cat. no. A7058). Following washing with Tris-buffered saline containing Tween 20 (Thermo Fisher Scientific, Waltham, MA; cat. no. 15805428), the resulting membranes were imaged with a Bio-Rad ChemicDoc imager using the stain-free blot application and the optimal automatic exposure setting. Thrombin cleavage to remove the hexa-histidine tag was not employed at any stage in the purification process.

### LspA-globomycin complex LCP sample preparation

LCP containing ^13^C/^15^N-labeled LspA and globomycin was prepared using a similar procedure to that described above for gramicidin-LCP preparation (Fig. S1). The main difference between the two procedures was that LspA was available in a detergent-solubilized liquid form, whereas gramicidin was available as a dry solid. Dry monoolein was heated at 42°C until molten and was then transferred into a 100-*μ*L gas-tight Hamilton syringe. Aliquots of ^13^C/^15^N-labeled LspA at 40 mg/mL in 50 mM MES-NaOH pH 6.15, 150 mM NaCl, 30%(v/v) deuterated glycerol and 0.14%(w/v) FC-12 were thawed on ice and incubated for 30 min on ice with a 10-fold molar excess of globomycin (Sigma-Aldrich, Saint Louis, MO, USA; cat. no. G1424) before transferring to a second 100 *μ*L gas-tight Hamilton syringe. The lipid and protein were then combined at RT as detailed above (section gramicidin-LCP sample preparation). The coupler was kept attached to the syringe in which the sample was being stored and was used to transfer the LCP sample directly into a 4 mm Bruker ssNMR MAS rotor.

### Solid-state NMR

ssNMR measurements were made using an 800 MHz Bruker Avance III spectrometer (Bruker, Billerica, MA, USA) equipped with 4-mm HR-MAS dual inverse ^1^H/^13^C or 4 mm ^1^H/^13^C/^15^N CP-MAS probes (Bruker, Billerica, MA, USA) and operating at 800 MHz (19 T, 201.2 MHz ^13^C Larmor frequency). Rotors (4 mm; Bruker, Billerica, MA, USA) were filled with 50 *μ*L of LCP containing gramicidin or globomycin-bound ^13^C/^15^N-labeled LspA. Acquisition and processing parameters for all ssNMR spectroscopy experiments are described in Tables S1–S9. Experiments were conducted at MAS frequencies of 5, 7.5, 10, or 14.5 kHz and temperatures of 20°C, 10°C, or 0°C (see supporting figures and tables for experiment-specific information). ^13^C and ^1^H chemical shifts for LCP samples were directly referenced with respect to external liquid samples of tetramethylsilane in methanol and 2,2-dimethyl-2-silapentane-5-sulphonic acid in water, respectively (49). ^15^N chemical shifts for LCP samples were directly referenced with respect to external samples of powdered ^15^NH_4_Cl (50). Temperature calibration of the 4 mm ^1^H/^13^C/^15^N CP-MAS probe was performed by measuring the frequency difference between the resonances for the methyl and hydroxyl protons of methanol in the tetramethylsilane samples (49).

For ^13^C experiments, spectra were collected by direct excitation with nuclear Overhauser enhancement (NOE) (51) or via ^1^H-^13^C cross-polarization (CP) (52) using the 4-mm ^1^H/^13^C/^15^N CP-MAS probe with 750 *μ*s CP and high-power ^1^H decoupling during acquisition. Temperature was maintained at 20°C, 10°C, or 0°C using a cold air supply to correct for frictional sample heating. Spectra were processed and analyzed in TopSpin version 4.4.0. Signal-to-noise ratios (SNRs) were calculated using the TopSpin SiNo command (53). SiNo calculates the SNR of 1D spectra using the formula SNR = maxval/(2 × noise), where maxval is the highest intensity in the specified spectral region and noise is the average signal intensity in the specified noise region. ^1^H-^13^C and ^1^H-^15^N CP-MAS spectra were recorded at MAS frequencies of 5, 7.5, 10, or 14.5 kHz. A 90° ^1^H CP spinlock pulse was applied at a field strength of 50 kHz and the Hartmann-Hahn (54) condition was achieved by applying the following CP conditions: pulse length, 750 *μ*s; pulse frequency, 70 kHz on the ^1^H channel (linear ramp: 100%–80%); field strength, 50 kHz on the ^13^C and ^15^N channels. ^1^H decoupling at 70 kHz was performed during acquisition using a SPINAL-64 sequence (55) with recycle delays of 2–3 s.

Two-dimensional ^13^C–^13^C cross-polarization dipolar assisted rotational resonance (CP-DARR) experiments (56) were conducted at 5 kHz MAS. During the initial 0.75 ms CP period, the ^1^H amplitude was linearly ramped from 80% to 100%. During the ^13^C chemical shift evolution period in the indirect dimension, continuous wave heteronuclear ^1^H-^13^C decoupling was used. ^1^H decoupling at 70 kHz was performed during evolution and signal acquisition periods using a SPINAL-64 sequence (55). Spectra were acquired using DARR mixing times of 400 ms. Recycle delays of 3 s were employed. On the ^13^C channel, the following field strengths were employed: 50 kHz for CP, and 50 kHz for *π*/2 pulses during the mixing period, respectively. CP-DARR experiments collected at 10 and 14.5 kHz MAS were performed with the MAS frequency parameter erroneously set to 5 kHz, which affected further processing and the appearance of the spectra (see legend to Fig. S8 for details). Saturation transfer difference experiments (57) were conducted as previously described (57,58,59,60). Briefly, 2D ^15^N-detected ^1^H MAS NMR spectra were recorded at an MAS frequency of 14.5 kHz and mixing time of 400 ms, and resonance attenuation was measured in response to saturating radiofrequency pulses (field strength, 0–2 kHz) consisting of 20 Gaussian-shaped 50 ms pulses. The saturation frequency was set to the amide region of the protein (8.55 ppm). The attenuation of the lipid methylene signal (1.1 ppm), defined as resonance amplitude recorded without saturation divided by the amplitude with saturation, was followed as an indicator of magnetization transfer to lipid. A total of 512 scans with a recycle delay of 2 s were acquired at 10°C. ^1^H decoupling at 70 kHz was performed during evolution and signal acquisition periods using a SPINAL-64 sequence (55).

In the ^15^N-^13^C^*α*^ SPECIFIC CP experiments, the spectral width and acquisition time were 219.89 ppm and 23.14 ms in the direct dimension, and 740.0 ppm and 1.07 ms in the indirect dimension. During the initial 0.75 ms CP period, the ^1^H amplitude was linearly ramped from 80% to 100%. During the ^15^N chemical shift evolution period, continuous-wave heteronuclear ^1^H-^15^N decoupling was used. The recycle delay was set to 1.5 s. The ^15^N-^13^C^*α*^ transfer was achieved by SPECIFIC CP (61) in which magnetization was transferred from ^1^H to ^15^N of the LspA backbone amides via CP and then selectively transferred via CP to the LspA backbone ^13^C^*α*^, with the ^15^N amplitude being linearly ramped from 80% to 100%. The CP mixing time was 6 ms. The heteronuclear decoupling field strength was kept at 50 kHz for the duration of the experiment. The ^15^N frequency was centered at 119 ppm; the ^13^C frequency was centered at 53 ppm. The ^15^N to ^13^C^*α*^ transfer used a tangent adiabatic ramp on the ^13^C channel (54). One tangential sweep of 8 kHz was carried out with a contact time of 3 ms. ^1^H decoupling at 70 kHz was performed during evolution and signal acquisition periods using a SPINAL-64 sequence (55).

### Solution NMR

Solution-state NMR experiments were conducted with ^15^N-, or ^2^H/^13^C/^15^N-labeled LspA in 50 mM MES-NaOH pH 6.15, 150 mM NaCl, and 0.14%(w/v) FC-12 at 50 to 100 *μ*M protein before and after the addition of a 10-fold molar excess of globomycin (Sigma-Aldrich, Saint Louis, MO, USA; cat. no. G1424). Samples contained 10% (v/v) D_2_O, which was used to provide the lock signal. ^1^H-^15^N heteronuclear single quantum coherence (HSQC) (43,62) and transverse relaxation-optimized spectroscopy (TROSY) (43,63) experiments were conducted on an 800-MHz Bruker Avance III spectrometer (Bruker, Billerica, MA, USA) equipped with a 5 mm TCI CryoProbe (Bruker, Billerica, MA, USA) operating at 25°C, 35°C, or 45°C. Experimental details and parameters for all solution NMR spectroscopy experiments are described in Tables S10 and S11. HSQC spectra were collected with 2048 × 256 total points and 96 scans. TROSY spectra were collected 2048 × 256 total points and 128 scans. ^1^H and ^15^N chemical shifts were referenced indirectly to 2,2-dimethyl-2-silapentane-5-sulphonic acid and ammonia, respectively, with comparison to the known chemical shift of water (64). Temperature calibrations were performed by measuring the frequency difference between the resonances for methyl and hydroxyl protons of methanol (49). Spectra were processed and analyzed in Bruker TopSpin (version 4.4.0). Cross-peak linewidths were determined via analytical parabolic fits in CCPN (65).

### FRET-based LspA activity assay

A fluorescence resonance energy transfer (FRET)-based assay was used to monitor the enzymatic activity of ^13^C/^15^N-labeled and unlabeled LspA using a synthetic FRET-labeled lipopeptide substrate (6). The assay buffer contained 100 mM MES-NaOH pH 5.6, 150 mM NaCl, 80 *μ*M FRET lipopeptide substrate, and 0.05%(w/v) lauryl maltose neopentyl glycol in a reaction volume of 50 *μ*L. LspA in buffer C was diluted to 0.1 *μ*M in the assay buffer before running activity assays. To demonstrate inhibition, 0.1 *μ*M enzyme was incubated with 1 *μ*M globomycin for 30 min on ice before running the FRET assay. Reaction progress was monitored by fluorescence (Ex/Em, 320 nm/420 nm) for 40 min at 37°C in a SpectraMax M2e plate reader (Molecular Devices, San Jose, CA, USA).

### In meso crystallization of globomycin-bound ^13^C/^15^N-labeled LspA

In meso crystallization trials were set up by transferring 50 nL of the protein-laden mesophase onto a siliconized 96-well glass crystallization plate, which was then covered with 800 nL of precipitant solution using in meso robots (23). The crystallization plates were prepared in-house by first silanizing 1 mm-thick 127.8 × 85.5 mm glass base plates (Marienfield, Lauda-Königshofen, Germany; cat. no. 1523127090) using rain-X glass water repellent (Kraco Car Care International, Liverpool, UK; cat. no. 88199500), widely available from hardware stores and garages. To the silanized plates, 77 × 112 mm (Saunders, Lombard, IL, USA) spacers with 6 mm holes were affixed. After setting in meso crystallization boluses, plates were sealed with 77 × 112 mm glass cover plates (Marienfield, Lauda-Königshofen, Germany; cat. no. 01029990911) and incubated at 20°C in a Rock Imager (Formulatrix, Bedford, MA, USA). Crystals were obtained using precipitant solutions containing 100 mM MES pH 5.6–6.0, 35%–43% (v/v) PEG400 and 60–100 mM ammonium phosphate monobasic. Plate-shaped crystals appeared after 1–3 days and continued to grow reaching dimensions of ca. 3 × 30 × 80 *μ*m^3^ in 2 weeks. All crystals were tested for diffraction at the synchrotron and generally displayed diffraction to approximately 3 Å resolution. The dataset used for structure determination was collected from a crystal grown at 100 mM MES pH 5.6, 40% (v/v) PEG400 and 100 mM ammonium phosphate monobasic. Crystals were harvested by opening the wells with a tungsten carbide glass-cutting tool, removing crystals with a minimum of adhering mesophase using Dual Thickness MicroLoops LD cryoloops (MiTeGen, Lansing, NY, USA) followed by snap-cooling in liquid nitrogen without added cryoprotectant.

### X-ray diffraction data collection and structure determination

X-ray diffraction experiments were performed at the protein crystallography beamline X06SA-PXI and X10SA-PXII, Swiss Light Source (SLS), Villigen-PSI, Switzerland, and I24, Diamond Light Source, Didcot, UK. Final data were collected at the SLS with a 10 × 10 *μ*m^2^ microfocused X-ray beam at 12.398 keV (1 Å) and 100 K using SLS data acquisition software suites (DA+) (66). Continuous grid scans were used to locate crystals in cryogenically cooled mesophase samples (67). Data were collected in steps of 0.2° at 0.1 s per step using the EIGER 16 M detector operated in continuous/shutterless data collection mode. A 120° dataset, recorded at a flux of 7 × 10^11^ photons s^−1^, was processed using autoPROC/STARANISO (68,69) followed by scaling and merging with *XSCALE* (70) (Table S12). Molecular replacement was used for phasing the structure (PDB: 5DIR) (5) as the initial searching model and a solution was obtained using Phaser (71). The coordinates for globomycin (BIRD: PRD_002257) (5,6) and monoolein were added to the model and iterative cycles of model building in COOT (72) and refinement in phenix.refine (73) were performed until no further improvements in the R_free_ or electron density were obtained. After validation in MolProbity (74), refined coordinates and structure factors were deposited in the RCSB (PDB: 9EMZ) (Table S12).

## Results and discussion

### ssNMR of gramicidin in LCP

The gramicidin-laden LCP sample used in this study had a peptide-to-lipid molar ratio of 1:20. This is the upper limit of the LCP’s carrying capacity for gramicidin. At higher concentrations, the cubic mesophase destabilizes and transitions to the inverted hexagonal phase (40). Parenthetically, we note that a 1:20 molar ratio corresponds to using an aqueous solution with a peptide concentration of ∼0.4 g/mL to make the peptide-laden LCP by the conventional method of mixing detergent-solubilized peptide with molten lipid (see Calculation S1) (23). The gramicidin-LCP samples used here and in other work (40,41,42) constitute, to our knowledge, the highest peptide loadings of the LCP reported so far. These high loadings are straightforward with transmembrane peptides, which may be prepared as dry solids and combined directly with MAG lipids before LCP formation. This strategy has been employed with other transmembrane peptides to yield loadings of the LCP significantly higher than is generally achievable when working with membrane proteins prepared in detergent-containing solutions (7,34). For membrane protein targets prepared in detergent-containing buffer, it is more common to combine the protein solution at 20–50 mg protein/mL with MAG lipid to generate protein-laden LCP for in meso crystallization trials (4,23,75).

**Figure 2:**
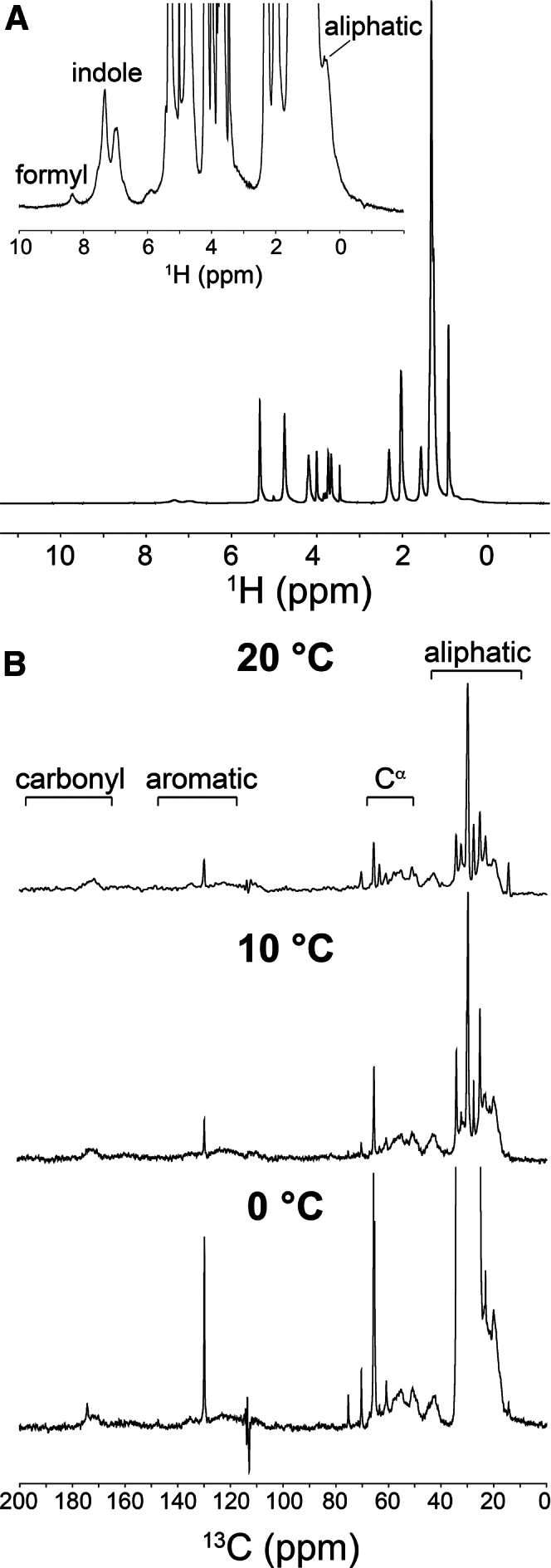
^1^H and ^13^C MAS ssNMR spectra of gramicidin-laden LCP. (*A*) ^1^H NMR spectrum of gramicidin-laden LCP recorded at 5 kHz and 20°C using the 4 mm HR-MAS dual inverse ^1^H/^13^C probe. A region of the spectrum with intensity scaled up 64-fold is included as an inset with characteristic gramicidin peaks labeled. (*B*) ^13^C CP-MAS NMR spectra of gramicidin-laden LCP recorded at decreasing temperatures. Spectra were acquired at 7.5 kHz using the 4 mm ^1^H/^13^C/^15^N CP-MAS probe with 750 *μ*s CP and high-power ^1^H decoupling during acquisition. The spectral regions in which particular resonance types (aliphatic, aromatic, C^*α*^) are expected to occur are indicated.

Gramicidin-LCP preparations at the stated molar ratio of 1:20 have produced diffraction- and structure-quality in meso gramicidin crystals with various MAG host lipids (41,42). 1D ^1^H spectra collected on LCP at this high peptide loading using the 4 mm HR-MAS dual inverse ^1^H/^13^C probe at 5 kHz revealed characteristic gramicidin resonances among water and lipid peaks (Fig. 2 *A*). Based on the previously reported gramicidin assignments (38), peaks at ∼0.3, ∼6.7/7.1, and ∼8.1 ppm were attributed to protons in valine and leucine side chains, in the indole of tryptophan, and in the N-terminal formyl of gramicidin, respectively (Fig. 2 *A*). Signals from the *α*-carbon (C^*α*^) protons of the gramicidin backbone, anticipated in the 50 to 60 ppm region of the spectrum, were obscured by overlapping proton signals from lipid and water^22^ (The cubic phase is approximately equal parts monoolein and water. This translates to a concentration of ∼2 M lipid and 28 M water. The corresponding concentration of gramicidin in the cubic phase is 0.084 M (158 mg/mL, see Calculation S1)).

The exceptionally high gramicidin concentration tolerated by the LCP facilitated detection of peptide ^13^C resonances at natural abundance but only when high-power ^1^H-^13^C CP was used (Fig. 2 *B*). By contrast, direct excitation ^13^C spectra of gramicidin-LCP at 5 kHz and 20°C obtained using the 4 mm HR-MAS dual inverse ^1^H/^13^C probe with NOE sensitivity enhancement were dominated by signals from the host lipid (Fig. S5), as previously reported (76,77,78). Peaks were assigned to individual lipid carbons based on published NMR spectra for monoolein (79) (Fig. S5 A). Broad gramicidin resonances were discernible in the noise, mainly in the aromatic and aliphatic carbon regions. With CP-MAS, ^13^C resonances from gramicidin were detected with a resolution of ∼1 ppm (Fig. 2 *B*). This is comparable to the resolution observed in ssNMR spectra of gramicidin reconstituted in phosphatidylcholine bilayers (37).

CP in ssNMR experiments depends on heteronuclear dipolar interactions. Therefore, the proximity of various nuclei, as determined by the overall flexibility of a target molecule, contributes significantly to CP efficiency (80). CP efficiency is reduced with increasing molecular motions and by high MAS rates, which interfere with dipolar interactions. Reducing peptide and protein dynamics by lowering sample temperature is a well-established strategy for improving the CP-MAS ssNMR spectra of gramicidin (81) and other membrane protein targets (82,83,84). It must also be noted that a reduction in protein dynamics at low temperature can lead to SNR enhancement. For gramicidin ^13^C resonances, the SNR increased ∼five- to seven-fold depending on the carbon atom type, as shown in Fig. 2 *B* and quantified in Table 1.

**Table 1:** SNR data for ^13^C ssNMR spectra of gramicidin-laden LCP acquired at 7.5-kHz MAS at 20°C, 10°C, and 0°C

Sample temperature	20°C	10°C	0°C
C^*α*^ signal frequency	65.1 ppm		
SNR^a^	20	38	144
SNR fold improvement^b^	1	1.9	7.2
Aromatic signal frequency	129.8 ppm		
SNR^a^	12	15	61
SNR fold improvement^b^	1	1.3	5.1
Carbonyl signal frequency	175.2 ppm		
SNR^a^	3.5	4.9	21
SNR fold improvement^b^	1	1.4	6.0

a SNR measurements were made using the SiNo plugin in Bruker TopSpin. For each SNR calculation, the noise level was set as that recorded in the 87–106 ppm region of the spectrum.

b Fold improvement in SNR is calculated relative to that at 20°C.

Below ∼17°C, the cubic phase formed by monoolein is in an undercooled state (85). This property is exploited in the crystallization screening of membrane proteins, which can be carried out over a range of temperatures, typically down to 4°C (86). The undercooled metastable state is long lived. This was observed in the current study where the sample remained in the cubic phase while NMR data were collected at 10°C and 0°C over the period of about a week. The sample remained optically transparent throughout, indicating that the LCP integrity was not compromised. Therefore, data collection for protracted periods of time with membrane protein-laden LCP samples should be possible over a range of temperatures. Such measurements may prove useful for time-dependent studies of protein stability in membrane-like environments (87).

### ssNMR of the LspA-globomycin complex in the LCP

The complex used in this study was prepared by incubating detergent-solubilized ^13^C/^15^N-labeled LspA with a 10-fold molar excess of globomycin for 30 min on ice. Reconstitution of the complex into the cubic mesophase was carried out by the traditional twin-syringe mixing method (Fig. S1) (23). Fifty microliters of the protein-laden mesophase, representing 1 mg of LspA, were transferred into the 4-mm ^1^H/^13^C/^15^N CP-MAS probe for ssNMR measurements.

For quality-control purposes, we recapitulated the previously described in meso LspA-globomycin co-crystal structure determination and *in vitro* activity assays (5,6) with the ^13^C/^15^N-labeled protein. In meso crystallization trials with the doubly labeled enzyme produced crystals and an X-ray diffraction structure to 3 Å resolution with a minimum of optimization (Table S12). As expected, the structures of the labeled and unlabeled protein are virtually identical (Fig. S11) with a root-mean-square deviation of 0.36 Å for backbone C^*α*^ atoms. Furthermore, LspA peptidase activity was unaffected, and the enzyme was inhibited by globomycin as judged by the FRET progress curves (Fig. S12). This supports the view that the ^13^C/^15^N-labeled LspA preparation used in the ssNMR experiments was properly folded and enzymatically active.

**Figure 3:**
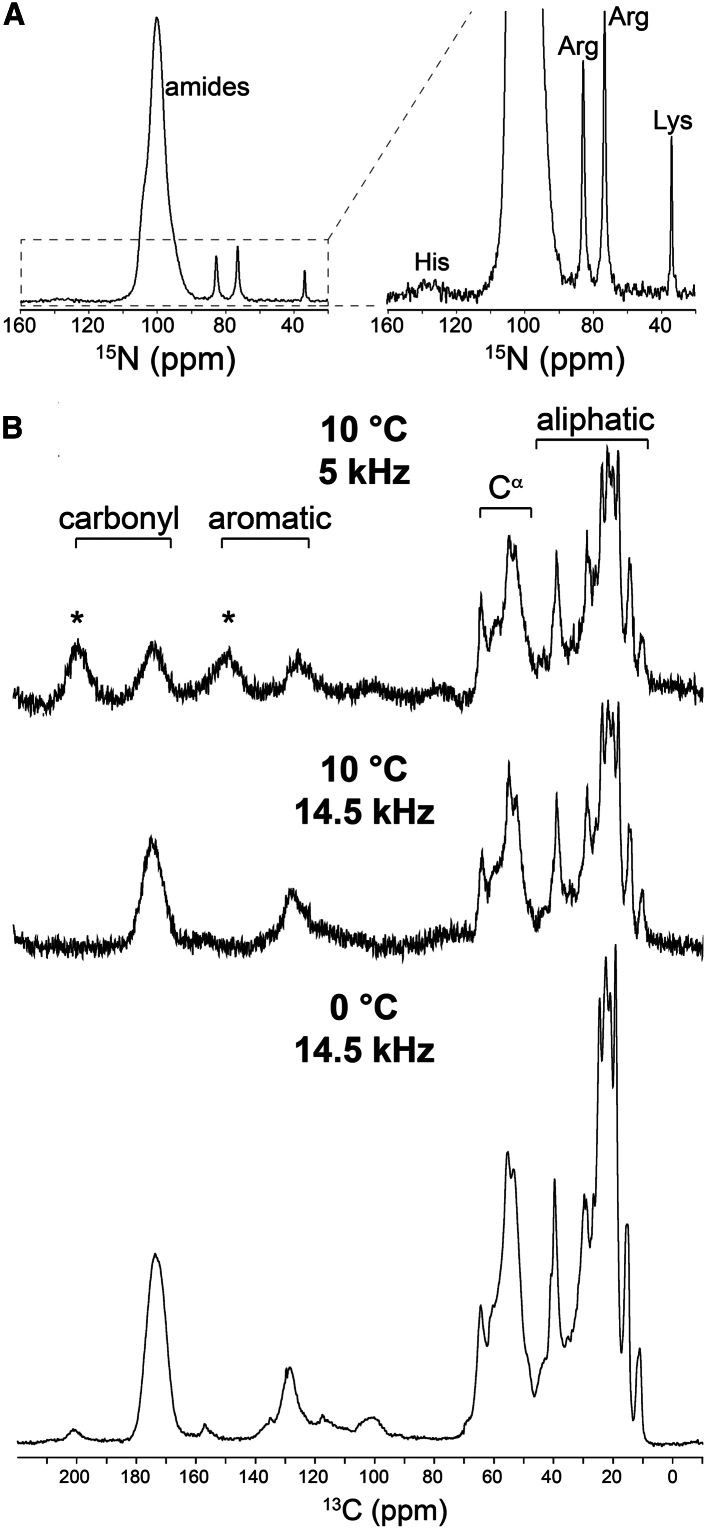
^15^N and ^13^C MAS ssNMR spectra of the LspA-globomycin complex in the LCP. (*A*) 1D ^15^N and (*B*) 1D ^13^C CP-MAS NMR spectra of the ^13^C/^15^N-labeled LspA-globomycin complex in the LCP recorded using the 4 mm ^1^H/^13^C/^15^N CP-MAS probe. The ^15^N CP-MAS spectrum was recorded at 10°C and 10 kHz. Signal intensity for each 1D ^13^C CP-MAS NMR spectrum collected at 10°C has been scaled up 16-fold. Spinning side bands, where visible, are indicated by an asterisk.

1D ^15^N spectra were acquired at 10°C and a MAS frequency of 10 kHz (Fig. 3 *A*). An intense signal for backbone amides was observed, along with well-resolved peaks for histidine, lysine, and arginine side chains. The spectral resolution and SNR are on par with other reported ^15^N ssNMR spectra for membrane proteins in lipid bilayers (10,11,12,13,14) and established membrane mimetics (13,14,15,16,17,18,19,20,21,22). As observed for ^13^C ssNMR data collection with gramicidin (Fig. 2 *B*), CP was required to resolve ^13^C resonances from LspA. By contrast, direct excitation ^13^C ssNMR experiments at 5 kHz MAS yielded peaks for monoolein alone, whereas protein resonances were mostly broadened beyond detection (Fig. S6). With CP and high-power ^1^H decoupling during acquisition, LspA ^13^C resonances were detected (Fig. 3 *B*). At 10°C and 5 kHz MAS, signals for aliphatic and C^*α*^ carbons were resolved, whereas aromatic and carbonyl resonances were difficult to discern from the noise. Increasing the spinning frequency from 5 to 14.5 kHz improved ^13^C SNR ∼two-fold, particularly in the aromatic and carbonyl regions (Fig. 3 *B*; Table 2). SNR in the C^*α*^ region was largely unchanged. LspA ^13^C spectra with maximal SNR were obtained at 0°C and 14.5 kHz MAS (Fig. 3 *B*). This is consistent with the results obtained for gramicidin at lower temperatures (Fig. 2 *B*; Table 1). The most notable improvement was observed in the carbonyl region of the spectrum, which displayed an ∼20-fold improvement in SNR with respect to spectra recorded at 10°C and 5 kHz MAS (Table 2).

**Table 2:** SNR data for ^13^C ssNMR spectra of LspA-laden LCP

MAS frequency	5 kHz	14.5 kHz	14.5 kHz
Sample temperature	10°C	10°C	0°C
C^*α*^ signal frequency	–	64.4 ppm	–
SNR^a^	10.2	11.3	71.9
SNR fold improvement^b^	1	1.1	7
Aromatic signal frequency	–	128.5 ppm	–
SNR^a^	12	23.2	40
SNR fold improvement^b^	1	1.9	3.3
Carbonyl signal frequency	–	173.8 ppm	–
SNR^a^	4.9	9.6	98.9
SNR fold improvement^b^	1	2	20.2

a SNR measurements were made using the SiNo plugin in Bruker TopSpin. For each SNR calculation, the noise level was set as that recorded in the 74–92 ppm region of the spectrum.

b Fold improvement in SNR is calculated relative to that at 5 kHz and 10°C.

**Figure 4:**
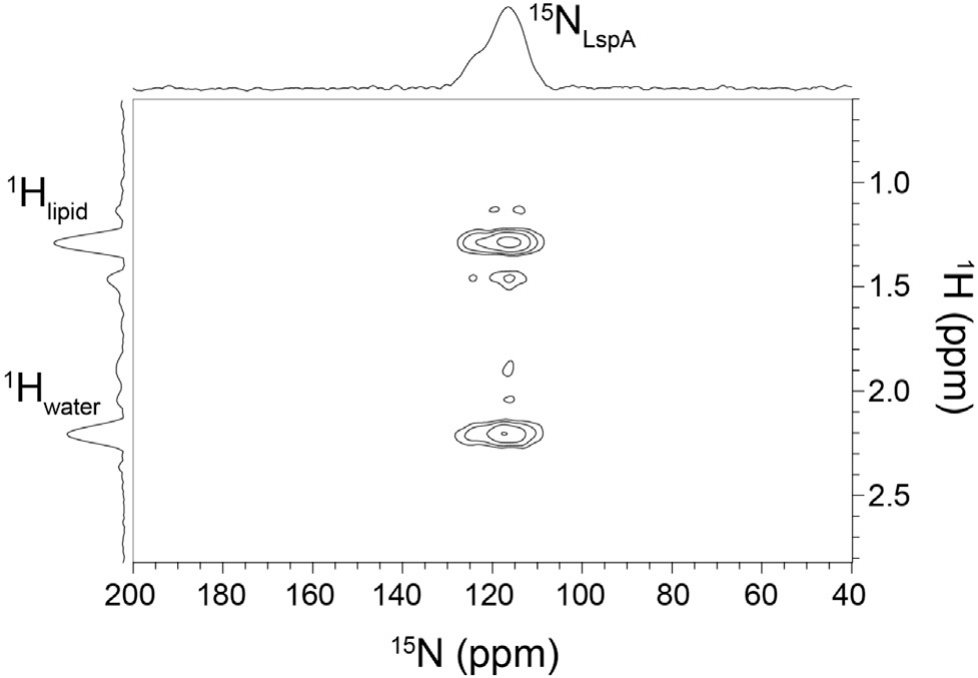
2D ^15^N-detected ^1^H saturation transfer difference spectrum of the LspA-globomycin complex in LCP collected at 10°C and a MAS frequency of 14.5 kHz using the 4 mm ^1^H/^13^C/^15^N CP-MAS probe with a mixing time of 400 ms. 128 T1 increments were acquired. 1D ^15^N (protein) and ^1^H (lipid and water) positive projections are shown above and to the left of the 2D spectrum. The lipid protons contributing to the signal at ∼1.1 ppm are those from the acyl chain methylene groups of monoolein.

Interactions between LspA and the lipid and water components of the cubic phase were investigated via a saturation transfer difference (57,58,59,60) experiment (Fig. 4). Here, the magnetization of lipid and water protons was transferred to the protons of the enzyme by way of an NOE sequence followed by CP transfer to directly bonded ^15^N nuclei of amides in the protein backbone. The resulting spectrum revealed two cross-peaks formed as a result of direct interactions of LspA with monoolein and water. The ^1^H_water_-^15^N_LspA_ and ^1^H_lipid_-^15^N_LspA_ cross-peaks were of similar intensity, suggesting that LspA molecules are exposed to both components of the mesophase. Protein aggregation may occur within the LCP. Indeed, self-association, as in nucleation, is a pre-requisite for crystal growth by the in meso method. However, this result indicates that LspA interactions with the lipid and water constituents of the mesophase are mainly unencumbered by factors such as protein-protein aggregation or self-association. Double CP experiments involving magnetization transfer from ^1^H to ^15^N of LspA backbone amides and then selectively to backbone ^13^C^*α*^ nuclei (61) were also performed (Fig. S7). The ^13^C^*α*^ nuclei were sufficiently excited to produce a broad cross-peak despite the SNR of the spectrum being low. This experiment demonstrates that the type of complex multi-CP experiments required for ssNMR assignment and structure elucidation are feasible using LCP samples containing labeled membrane proteins.

2D CP-DARR ssNMR experiments were performed to probe inter- and intra-molecular interactions of the enzyme reconstituted into the membrane of the cubic mesophase. CP-DARR spectra collected at 10°C and 5 kHz MAS revealed cross-peaks of weak intensity (Fig. S8, top panel). CP-DARR signal intensity, SNR, linewidths, and resolution improved by increasing the spinning frequency from 5 to 10 kHz (Fig. S8, middle panel). At 10 kHz (10°C) and 14.5 kHz (0°C) MAS (Fig. S8, bottom panel), broad cross-peaks in the aliphatic and C^*α*^-carbonyl correlation regions were discernible from the spectral noise. Under these conditions, resonances in the C^*α*^–C^*β*/*γ*^ regions were of higher intensity, albeit cross-peak resolution was poor. Of the conditions tested, the best CP-DARR spectrum (most cross-peaks, highest SNR, narrowest linewidths) was achieved at 0°C and 14.5 kHz MAS (Fig. S8, bottom panel), in line with the 1D ^13^C experiments (Fig. 3 *B*). The improvement in resolution for C^*α*^–C^*β*/*γ*^ cross-peaks is stark (Fig. S8). Cross-peaks in the C^*α*^-carbonyl regions remained broad and of weak intensity even at the higher spinning frequency (14.5 kHz) and lower temperature (0°C) (Fig. S8, bottom panel). These features possibly arise from an attenuation of LspA backbone dynamics as a result of globomycin-induced rigidification (43,44) and/or immobilization of the protein in the bilayer of the mesophase. However, there are issues associated with the 10 and 14.5 kHz MAS spectra because incorrect acquisition parameters were used inadvertently for data collection (see Fig. S8 for full details). Nonetheless, we include the data here as a proof of concept to show that meaningful CP-DARR measurements can be made with membrane protein-laden LCP samples.

### LspA solution NMR

**Figure 5:**
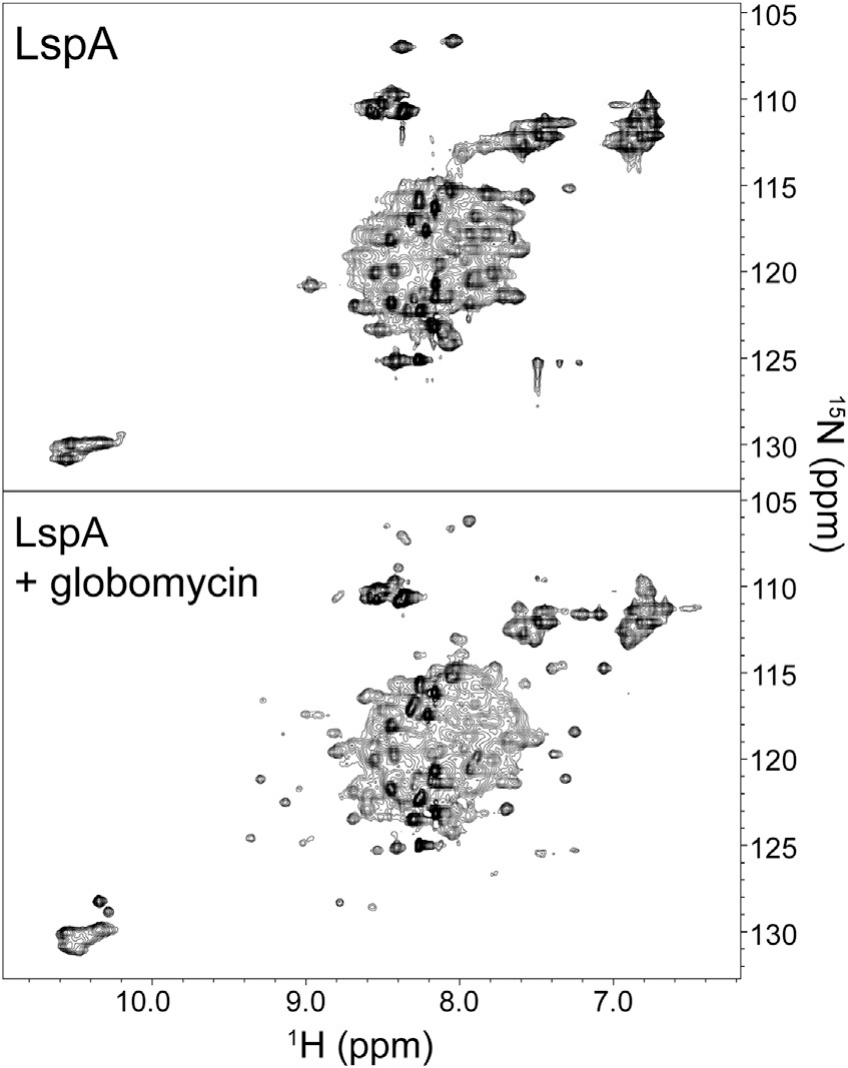
^1^H-^15^N HSQC spectra of ^15^N-labeled LspA in FC-12 micelles recorded at 800 MHz using a TCI CryoProbe. Spectra for globomycin-free and globomycin-bound LspA were collected at 25°C.

The effects of globomycin complexation on LspA structure were further investigated via solution-state NMR experiments. Solution ^1^H-^15^N HSQC and TROSY spectra of the globomycin-free and globomycin-bound forms of LspA were acquired using singly and triply labeled protein in FC-12 micelles (Figs. 5, S9, and S10). ^1^H-^15^N HSQCs collected with *in vivo*-expressed ^15^N-labeled LspA at 100 *μ*M protein and 25°C revealed considerable resonance overlap (Fig. 5, top panel). The extent of overlap was akin to that observed by Dötsch and co-workers for samples of ^15^N-labeled LspA reconstituted in FC-12 or DDM micelles (43). Only a few nonoverlapping cross-peaks were observed for LspA in its globomycin-free state. The addition of a 10-fold molar excess of globomycin produced large changes in the spectrum (Fig. 5, bottom panel). A clustered region of overlapping cross-peaks remained in the ^1^H 7.5–8.8 ppm, ^15^N 115–125 ppm region. However, a total of ∼25 nonoverlapping cross-peaks were detected, constituting an ∼four-fold improvement in the number of resolved cross-peaks compared to the globomycin-free spectrum. Similar improvements that include multiple additional resonances accompanied by wider peak dispersion have previously been reported for LspA upon complexation with globomycin (43). To probe the thermal stability of the LspA-globomycin complex at temperatures above 25°C, additional ^1^H-^15^N HSQC spectra for the ^15^N-labeled sample were collected at 35°C and 45°C (Fig. S9). A spectrum-wide downfield shift of the ^1^H-^15^N resonances occurred when temperature was increased from 25°C to 35°C on to 45°C. However, as judged by an overall similarity of the spectra, the elevated temperature did not significantly affect LspA tertiary structure. Previously reported solution NMRs of globomycin-bound LspA at 45°C are consistent with the enzyme-globomycin complex being thermally stable (43).

^1^H-^15^N TROSY experiments were conducted on cell-free-expressed ^2^H/^13^C/^15^N-labeled LspA at a concentration of 50 *μ*M protein (Fig. S10). Forty-four ^1^H-^15^N cross-peaks, with an average ^1^H^N^ linewidth of 17.4 ± 5 Hz, were detected for the globomycin-free protein, constituting approximately one-quarter of the possible ^1^H-^15^N resonances (Fig. S10, top panel; Table S13). These sharp resonances occurred in a narrow ^1^H 7.8- to 8.5-ppm, ^15^N 110- to 129-ppm region, indicative of a dynamic protein with limited tertiary structure (88). Compared to the HSQC experiments (Fig. 5, top panel), the resonance resolution was much improved for the triply labeled protein as expected for TROSY measurements carried out with protein incorporating deuterated amino acids (48). With the addition of a 10-fold molar excess of globomycin, LspA resonances broadened significantly as judged by the increased linewidths (Fig. S10, bottom panel; Table S14). Indeed, it was necessary to raise sample temperature from 25°C to 45°C to detect a measurable signal. The resulting cross-peak chemical shift dispersion for the globomycin-bound protein was wider than that for globomycin-free LspA. This is in line with the HSQC data (Fig. 5) and is consistent with the enzyme adopting a more ordered tertiary structure. ^1^H^N^ resonances for tryptophan indole side chains, undetectable in the globomycin-free spectrum, were observable upon complexation with globomycin at higher temperature (Fig. S10, bottom panel). Alongside well-resolved resonances, clusters of overlapping cross-peaks were observed in the ^1^H 8.0 to 8.5 ppm, ^15^N 115 to 124 ppm region. Despite sample temperature elevation, cross-peaks for the globomycin-bound protein were broader than for the free form with average ^1^H^N^ linewidths of 25.6 ± 5 Hz (Table S14). ^1^H-^15^N TROSY experiments carried out by Laguerre et al. with ^2^H/^15^N-labeled LspA-globomycin in FC-12 micelles yielded spectra with similar spectral overlap (43). Laguerre et al. subsequently succeeded in obtaining ^1^H-^15^N TROSY data for the LspA-globomycin complex of high spectral quality using a nanodisk-to-bicelle preparation of uniformly ^2^H/^13^C/^15^N-labeled LspA in which 98% of all possible ^1^H-^15^N resonances were assigned.

LspA solution NMR data corroborate the view that LspA undergoes considerable structural and dynamical rearrangements upon complexation with globomycin and the macrocyclic antibiotic, myxovirescin (5,6,43,44). These structural changes likely involve stabilization of the LspA backbone in a more rigid conformation. This proposed rigidification correlates well with our crystallization work that led to the structure determination of LspA-antibiotic complexes (5,6). By contrast, the ligand-free form of the enzyme has not yielded to crystallographic structure determination, possibly a consequence of its dynamic nature as suggested by the sharp resonances and narrow peak dispersion observed in the globomycin-free TROSY spectrum (Fig. S10, top panel). It is possible too that reconstitution into the lipid bilayer of the cubic mesophase, as a prelude to crystallization, contributes to the stabilization of these structural dynamics and to its in meso crystallizability. Noteworthy, in this regard, is the fact that LspA has not yielded to crystallization and structure determination by any technique other than the in meso method.

## Conclusions

Membrane mimetics are a staple of the membrane protein ssNMR field. Nanodiscs, bicelles, liposomes, liquid crystals, and oriented bilayers have been used with considerable success to generate ssNMR data for membrane proteins and peptides of different sizes, origins, and function (10,11,12,13,14,15,16,17,18,19,20,21,22). In this work, we investigated the cubic mesophase as a mimetic with which to perform ssNMR. Our findings show the cubic phase to be a useful mimetic that enables facile and homogenous reconstitution of membrane proteins and peptides into a bilayered membrane at high concentrations. Sample temperature and MAS frequency were identified as key variables for optimizing ssNMR measurements with protein-laden LCP samples (Figs. 2 *B* and 3 *B*; Tables 1 and 2). The capacity of the cubic mesophase to undercool in a metastable state enabled measurements at 0°C. The attendant improvement in SNR of the gramicidin and LspA ssNMR spectra (Figs. 2 *B* and 3 *B*) presumably arose from a combination of the lower measurement temperature and the small changes in mesophase microstructure that accompany temperature adjustment (85). It has been shown that the cubic phase can persist in an undercooled state down to at least −15°C (85). This feature might be exploited in future ssNMR work as a means for generating better-quality data and for use with thermally sensitive targets. Likewise, MAS frequencies above 14.5 kHz may prove beneficial for ssNMR of membrane proteins in the LCP. Of note is the fact that ^1^H-^13^C CP proved essential for the detection of protein resonances in ^13^C ssNMR measurements with LCP samples. This becomes apparent when spectra recorded using CP (Figs. 2 and 3 *B*) are compared with the spectra recorded using direct excitation (Figs. S5 and S6).

ssNMR spectra obtained with gramicidin in the cubic mesophase were comparable to those recorded with the peptide reconstituted into planar lipid bilayers (37) in terms of SNR and signal resolution. This suggests that in meso ssNMR is a convenient, rapid, user-friendly, and inexpensive alternative to oriented bilayers as a system in which to study peptides and proteins with a single membrane-spanning helix. Until recently, solution NMR was the principal method for investigating bitopic membrane protein structure (33,34,35,36). However, ssNMR (89,90) and in meso crystallography (7,91,92,93) are growing in popularity for such endeavors. Given the complementary nature of the information forthcoming from NMR and crystallography, and the fact that both can now be carried out with the same cubic-phase membrane mimetic, a promising approach will be to apply the two methods in parallel to the same membrane protein target. Relatedly, the in meso crystallization method often produces showers of small membrane protein crystals that are unsuitable for traditional crystallography but that have found application in serial X-ray crystallography (94,95). It should be possible to use these directly in the cubic phase for ssNMR analysis as has been done with micro-crystalline samples of proteins and protein complexes (96,97).

The utility of the cubic mesophase as a mimetic for ssNMR of membrane proteins was tested with LspA, a relatively small integral membrane enzyme and a target for antibiotic development (5,6). 1D ^15^N and ^13^C LspA spectra were recorded (Fig. 3) that had comparable resolution and SNR to those reported in the literature for other membrane proteins (10,11,12,13,14,15,16,17,18,19,20,21,22). As was observed in the ssNMR spectra of gramicidin in the LCP, the resolution and SNR of the LspA resonances improved upon lowering temperature and increasing sample spinning frequency (Fig. 3 *B*; Table 2). This finding is encouraging given that the LspA sample used in these studies contained just 1 mg of ^13^C/^15^N-labeled protein. ssNMR experiments conducted on LspA provided information on the dynamics of the protein in the membrane and on its interaction with the lipid and water components of the mesophase (Fig. 4).

Weak C^*α*^-carbonyl cross-peaks observed in CP-DARR spectra might suggest that the backbone of LspA in complex with globomycin is rigid, in line with the in meso crystal structures (5,6) and solution NMR data (43) reported to date. Further details on LspA structure and dynamics in the globomycin-free and globomycin-bound states were gleaned from solution NMR experiments. The previously described ligand-induced rigidification of LspA (43,44) was recapitulated as shown by the increase in chemical shift dispersion (Fig. 5). Addition of antibiotic resulted in an ∼20% increase in the number of nonoverlapping cross-peaks observed in HSQC experiments (Fig. 5).

The original in meso LspA-globomycin crystal structure was reproduced using the ^13^C/^15^N-labeled protein (Fig. S11; Table S12). Negligible structural differences were observed between the labeled and unlabeled protein (Fig. S11). No change in LspA peptidase activity was recorded as judged by FRET (Fig. S12).

For many membrane proteins, such as G protein-coupled receptors (8,12,20,32), the preparation of samples at a sufficiently high concentration for crystallization and ssNMR experiments can be limited by aggregation and/or denaturation. The cubic phase, which has been shown in this study to work for both crystallization and ssNMR, has a high carrying capacity for membrane proteins and peptides. Accordingly, the mesophase should now be considered as an optional membrane mimetic for ssNMR studies of proteins that are thermally sensitive and/or prone to aggregation. These proof-of-concept experiments conducted with LspA indicate that the amount of labeled protein required for a successful series of LCP-based ssNMR experiments is on a par with the ∼1 mg required for an initial round of crystallization screening. This quantity of protein was reconstituted into LCP by combining a 40-mg/mL solution of ^13^C/^15^N-labeled and detergent-solubilized LspA with molten monoolein. The resulting in meso protein concentration was approximately 16 mg/mL. This level of loading for a membrane mimetic is generally higher than is achievable with other staples of the membrane-protein ssNMR field, including bilayers (10,11,12,13,14), nanodiscs (15,16), bicelles (17,18), and liposomes (20,21,22). However, we acknowledge that these established mimetics are generally considered more native-like membranous environments than LCP given that MAGs are not important components of native membranes. It should be noted, however, that the cubic mesophase is customizable in composition and microstructure by doping with endogenous lipids to increase its physiological relevance (4,23).

The gramicidin-LCP samples analyzed here by ssNMR were prepared at the upper limit of LCP loading at 20 mol % (40). The resulting in meso peptide concentration was approximately 158 mg/mL (Calculation S1). We previously surveyed the PDB for the different protein concentrations employed in generating protein-laden LCP for crystallization experiments and found numerous examples where starting protein solutions were above 50 mg/mL (75). The majority of in meso crystal structures, however, have been obtained using starting membrane protein solutions in the 10 to 50 mg/mL range, as employed here for ssNMR and in meso crystallization of LspA. Thus, users of the LCP ssNMR methodology have a wide range of protein loadings with which to prepare samples. In situations where the maximum accessible protein solution concentration is extremely low, the “cubicon” method (75) can be used to incrementally raise protein concentration in the cubic phase by using the mesophase itself as a concentrator.

## Acknowledgments

This research was supported by 10.13039/501100001602Science Foundation Ireland (16/IA/4435 and 22/FFP-A/10278 to M.C.), the 10.13039/100030692Intramural Research Program of the 10.13039/100000002National Institutes of Health (NIH) (K.G., O.S., and H.K.), the European Union’s 10.13039/501100007601Horizon 2020 Research and Innovation Program under the Marie-Skłodowska-Curie program (701647 to C.-Y.H.), and a 10.13039/501100001711Swiss National Science Foundation Early Postdoc Mobility Fellowship grant (P2BSP3_15254 to M.W.).

We thank the Swiss Light Source and the Diamond Light Source synchrotron facilities for X-ray beam time allocations and the staff at beamlines PXII (SLS) and I23 (DLS) for their help with data collection. We thank W. Teague for assistance with data retrieval and L. Cerofolini for consultation on data quality.

The reviewers of this manuscript provided a wealth of valuable information, advice, and encouragement that enormously improved the clarity, accuracy, and quality of the final document. We thank them for their patience and for the time and effort they devoted to reviewing the work. They went beyond the call of duty.

## Author contributions

M.C. conceived the study. C.B., J.B., and M.W. prepared the protein and LCP samples. J.B. carried out functional assays and in meso crystallization. C.-Y.H. did the X-ray diffraction data collection, processing, and crystal structure determination. H.K., O.S., and K.G. performed the NMR experiments. K.O.R. processed and analyzed the NMR data. K.G., K.O.R., and M.C. interpreted the data and K.O.R., M.W., and M.C. wrote the manuscript. All authors commented on and approved the final version of the manuscript.

## Declaration of interests

The authors declare no competing interests.

## Supporting material

Document S1. Figures S1–S12 and Tables S1–S14

Figure360. An author presentation of Figure 1

Document S2. Article plus supporting material
