# Supplementary material for: Solid-state NMR of membrane peptides and proteins in the lipid cubic phase
Source: Biophys J. 2025 Mar 20;124(9):1387–400. doi: 10.1016/j.bpj.2025.03.012 (PMC12256886; doi:10.1016/j.bpj.2025.03.012)
Supplement: Document S1. Figures S1–S12 and Tables S1–S14 [file mmc1.pdf]

Biophysical Journal, Volume *124*

Supplemental information

Solid-state NMR of membrane peptides and proteins in the lipid cubic phase

Kiefer O. Ramberg, Coilin Boland, Hamed Kooshapur, Olivier Soubias, Maciej Wiktor, Chia-Ying Huang, Jonathan Bailey, Klaus Gawrisch, and Martin Caffrey

Supplemental Figures

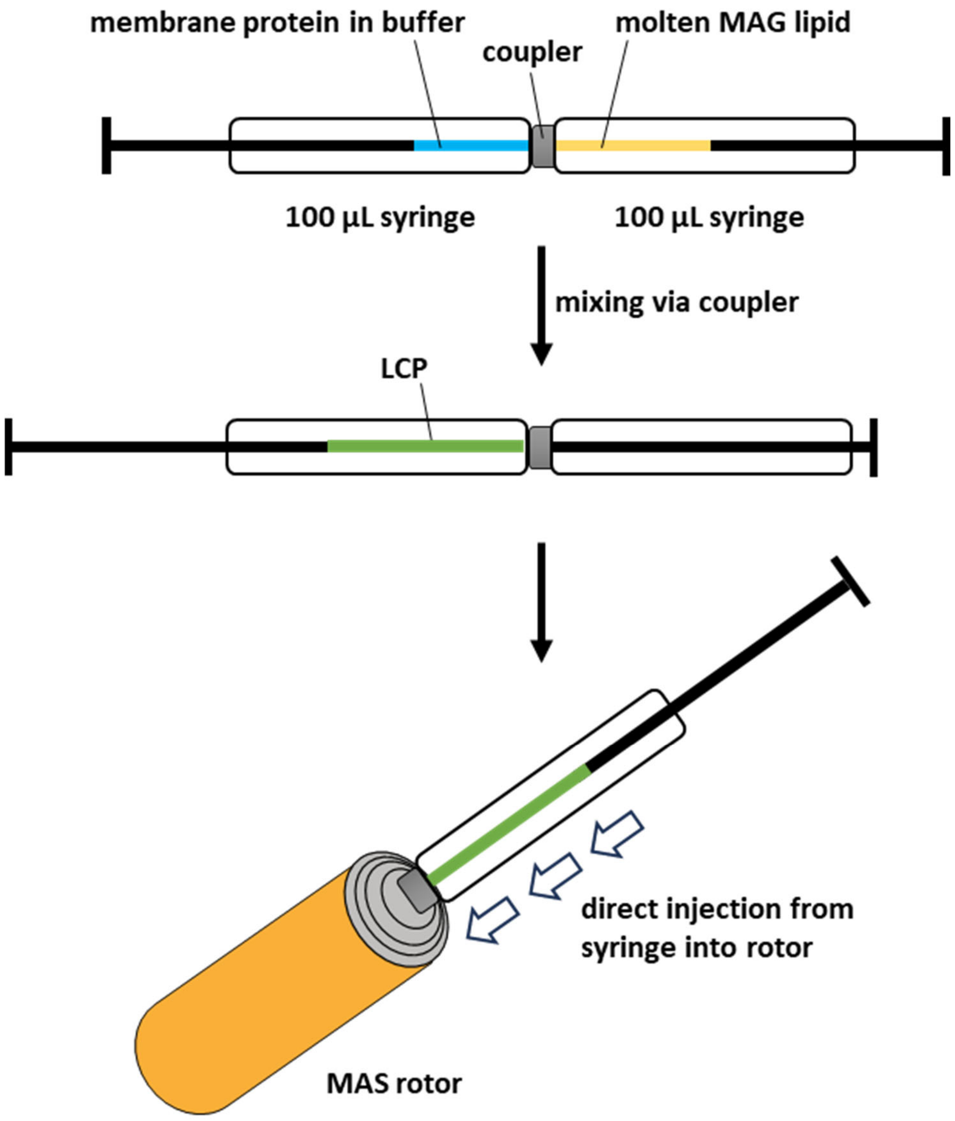

Figure S1. Preparation of membrane protein-laden LCP samples for ssNMR and loading into ssNMR rotors. Preparation of LspA-globomycin LCP samples was carried out by combining LspA (pre-equilibrated with a 10-fold molar excess of globomycin) at 40 mg/mL in 50 mM MES-NaOH pH 6.15, 150 mM NaCl, 30 %(v/v) deuterated glycerol and 0.14 %(w/v) FC-12 with molten monoolein at a 3:2 weight ratio of dry monoolein to protein solution as described previously (1). Preparation of gramicidin-laden LCP ssNMR samples was carried out using a similar protocol with the distinction that at the start of the process the syringe on the right contained a molten lipid/peptide mixture and the syringe on the left contained 25 mM sodium potassium phosphate pH 5.6. Both LCP preparations were transferred from the syringe into the ssNMR rotor via the syringe coupler.

A

MGSSHHHHHHSSGLVPR↓GSHMPDVDRFGRLPWLWITVLVFVLDQVSKAFFQAELSMYQQIV
VIPDLFSWTLAYNTGAAFSFLADSSGWQRWLFALIAIVVSASLVVWLKRLKKGETWLAIALA
LVLGGALGNLYDRMVLGHVVDFILVHWQNRWYFPAFNLADSAITVGAVMLALDMFRSKKSGE
AAHG

hexa-histidine tag　　LspA sequence　　↓ Cut site　　Thrombin protease site

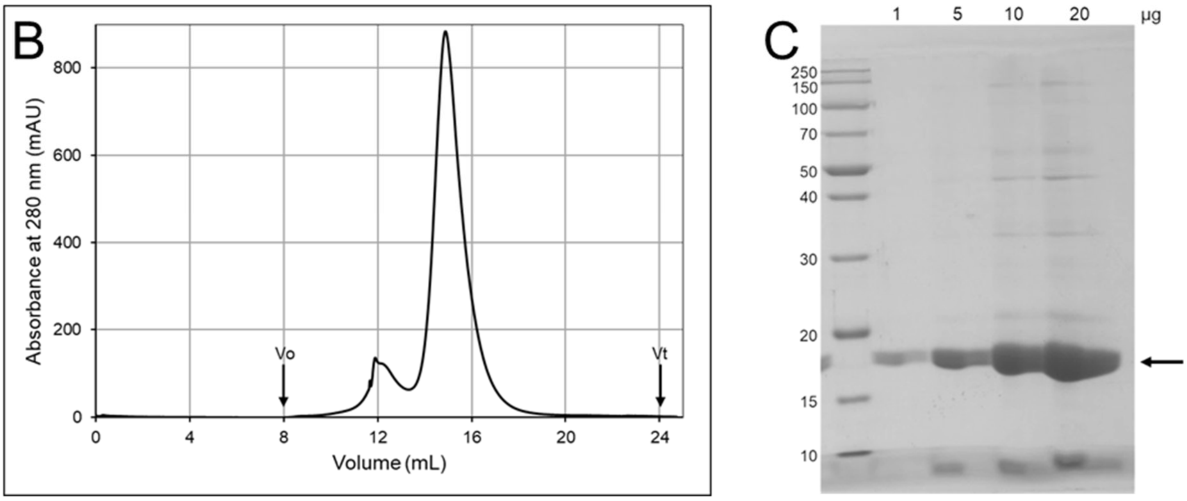

Figure S2. (A) LspA sequence (PDB ID 5DIR, UniProt Q9HVM5). Note that all protein preparations used in this work were not subjected to thrombin cleavage and remained hexa-histidine tagged. **(B)** Gel filtration chromatogram of *in vivo*-expressed ^{15}N-labelled LspA. Peak fractions eluting at 14.5 – 15.5 mL were pooled and concentrated to yield the final stock of ^{15}N-labelled LspA used for NMR experiments. Vo, void volume. Vt, total volume. **(C)** Coomassie-stained SDS-PAGE gel with loading series of *in vivo*-expressed ^{15}N-labelled LspA pooled and concentrated after gel filtration. A molecular weight ladder (PageRuler Unstained Broad Range Protein Ladder; Thermo Fisher Scientific, Waltham, MA, USA; cat. no. 26630) was run alongside the loading series samples. Molecular weights of the protein standards are reported in kDa. The horizontal arrow points to the band corresponding to monomeric LspA.

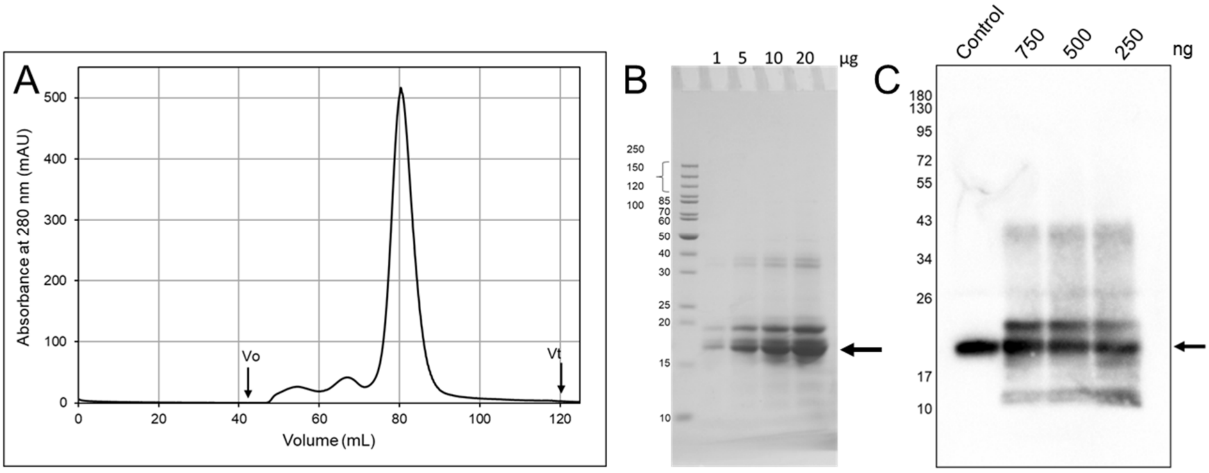

Figure S3. (A) Gel filtration chromatogram of $^{13}C/^{15}N$-labelled LspA. Vo, void volume. Vt, total volume. **(B)** SDS-PAGE (Coomassie-stained) gel with loading series of $^{13}C/^{15}N$-labelled LspA. A molecular weight ladder (PageRuler Unstained Broad Range Protein Ladder; Thermo Fisher Scientific, Waltham, MA, USA; cat. no. 26630) was run alongside the loading series samples. **(C)** SDS-PAGE (Western blot) with loading series for $^{13}C/^{15}N$-labelled LspA using anti-poly-Histidine antibody (Sigma-Aldrich, Saint Louis, MO, USA; cat. no. A7058). His-tagged LspA produced via *in vivo* expression in *E. coli* without isotope labelling is run alongside the $^{13}C/^{15}N$-labelled LspA samples as a control. The horizontal arrows point to the position on the gels/blots to which monomeric LspA usually migrates. A molecular weight ladder (PageRuler Prestained Protein Ladder; Thermo Fisher Scientific, Waltham, MA, USA; cat. no. 26619) was run alongside the loading series samples (not shown). Molecular weights of the protein standards in the molecular weight ladders are reported in kDa.

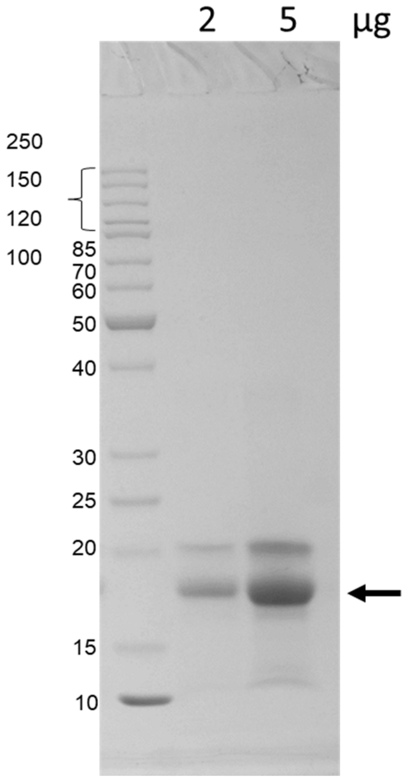

Figure S4. Coomassie-stained SDS-PAGE loading series for $^{2}H/^{13}C/^{15}N$-labelled LspA. The horizontal arrow points to the position on the gel to which monomeric LspA usually migrates. A molecular weight ladder (PageRuler Unstained Broad Range Protein Ladder; Thermo Fisher Scientific, Waltham, MA, USA; cat. no. 26630) was run alongside the loading series samples. Molecular weights of the protein standards are reported in kDa.

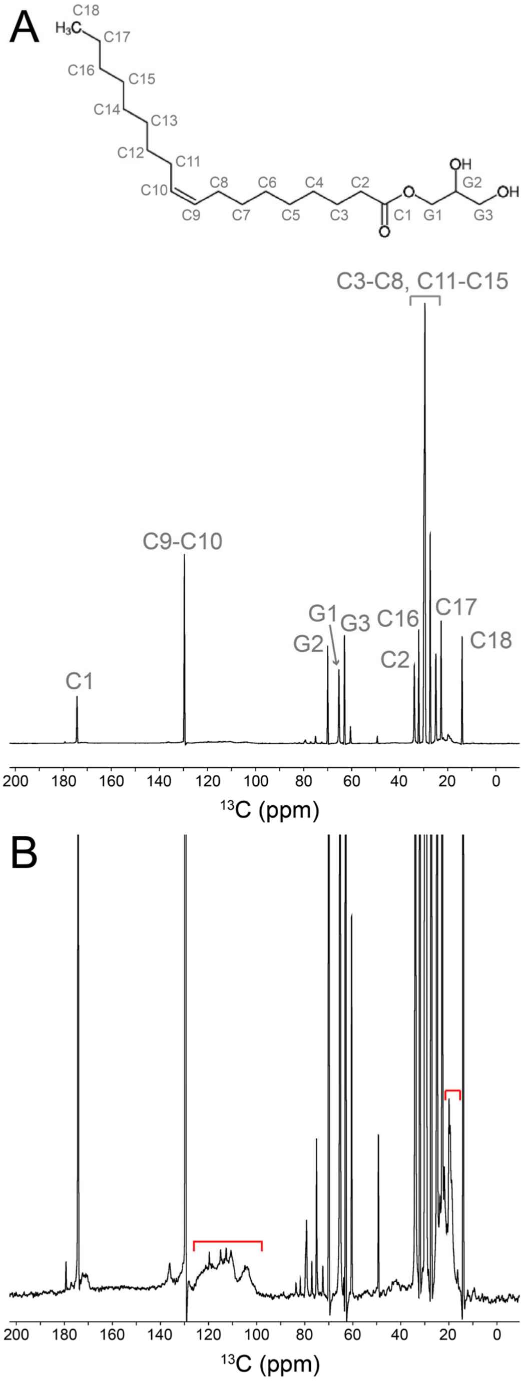

Figure S5. (A) Chemical structure of monoolein and direct excitation ^{13}C MAS NMR spectrum of gramicidin-laden LCP. Monoolein peaks, recorded with excellent resolution, were assigned to individual carbon atoms based on previously reported spectra for monoolein (2). The spectrum was acquired at 5 kHz MAS using the 4 mm HR-MAS dual inverse ^{1}H/^{13}C probe at 20 °C with NOE sensitivity enhancement. **(B)** Scale expansion of the spectrum in (A) highlighting broad gramicidin resonances (red brackets) discernible in the noise in the aromatic and aliphatic carbon regions. The amino acid C^{α} resonances are mostly broadened beyond detection.

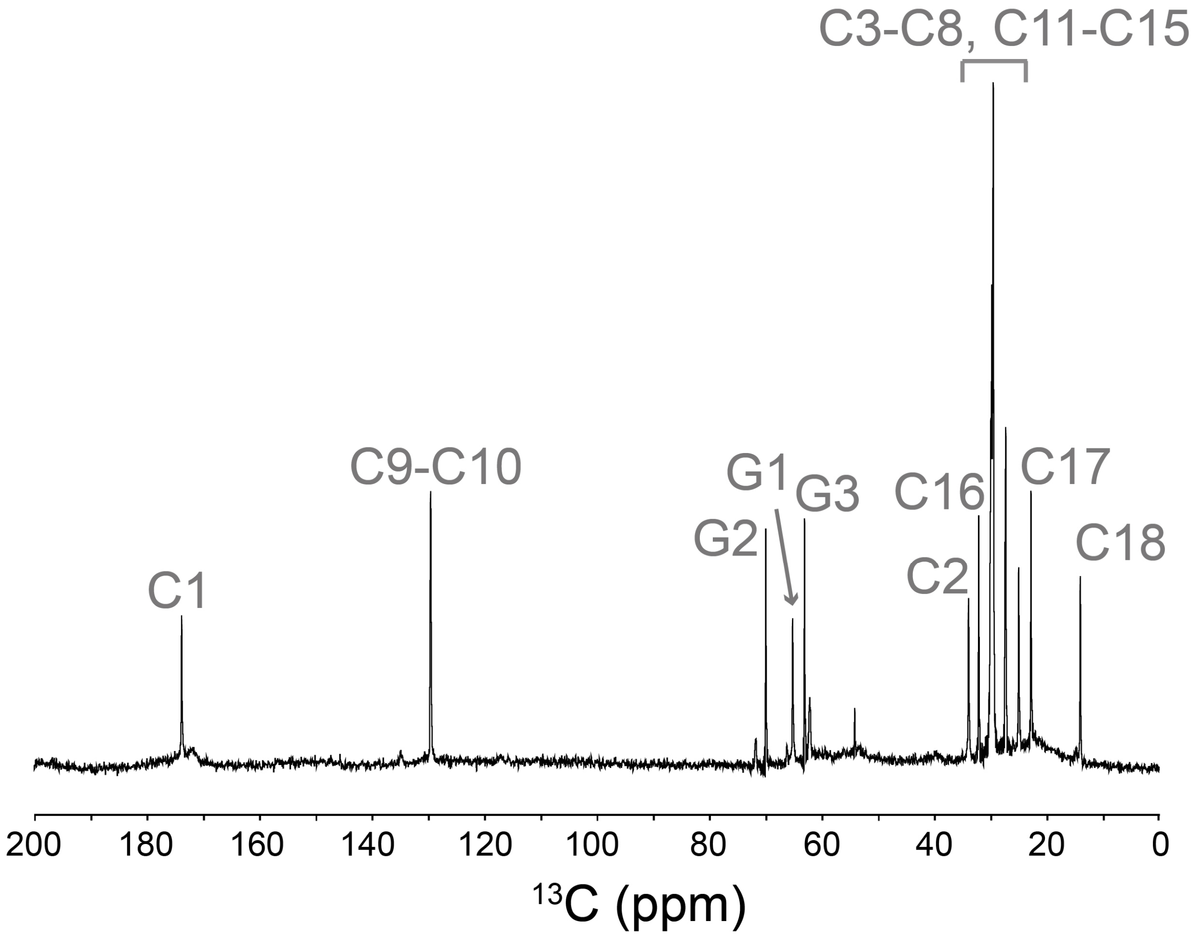

Figure S6. Direct excitation ^{13}C MAS NMR spectrum of LCP containing globomycin-bound LspA. Spectra were acquired at 5 kHz MAS using the 4 mm HR-MAS dual inverse $^{1}H/^{13}C$ probe at 20 °C with NOE sensitivity enhancement. Peaks for monoolein alone were observed and were assigned based on previously reported spectra for monoolein (2). For reference, the chemical structure of monoolein is shown in Figure S5.

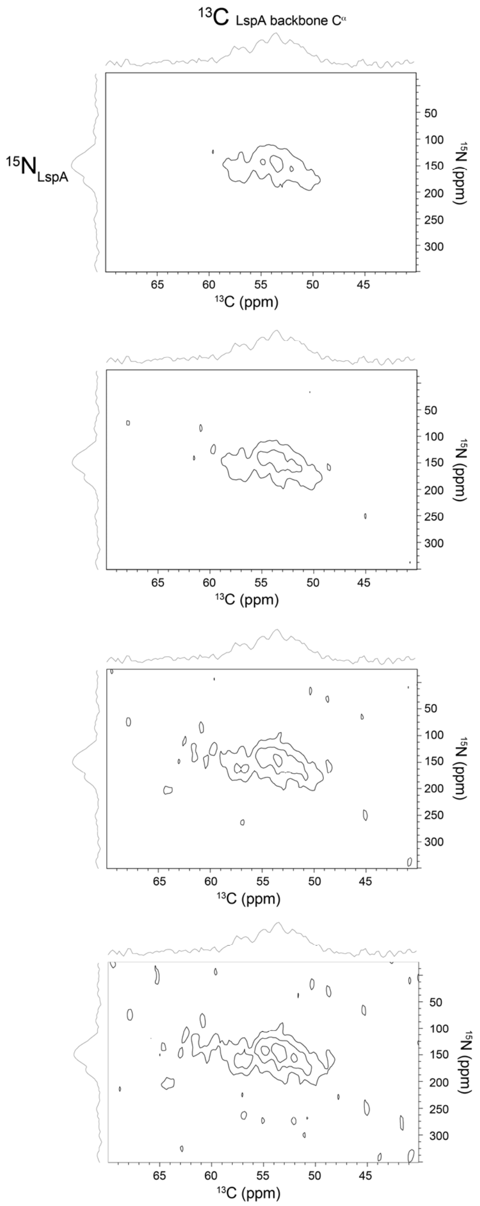

Figure S7. The 2D ^{15}N-^{13}C$^{\alpha}$ (NCA) ssNMR spectrum for globomycin-bound ^{13}C/^{15}N-labelled LspA in LCP shown at decreasing threshold levels. The threshold level is lowered two-fold in each panel from top to bottom to aid in visualizing the broad cross-peak. 1D ^{13}C (LspA backbone C$^{\alpha}$) and ^{15}N (LspA backbone amides) positive projection spectra are shown above and to the left of the 2D spectra, respectively. The experiment was conducted at 0 °C with MAS at 14.5 kHz using the 4-mm ^{1}H/^{13}C/^{15}N CPMAS probe. The spectral width and acquisition time were 219.89 ppm and 23.14 ms in the direct dimension (horizontal axis), and 740.0 ppm and 1.06 ms in the indirect dimension (vertical axis). The recycle delay was 1.5 s. NCA transfer was achieved when magnetisation was transferred from ^{1}H to ^{15}N of the LspA backbone amides via cross polarization and then selectively transferred to the LspA backbone ^{13}C$^{\alpha}$ using SPECIFIC CP (3).

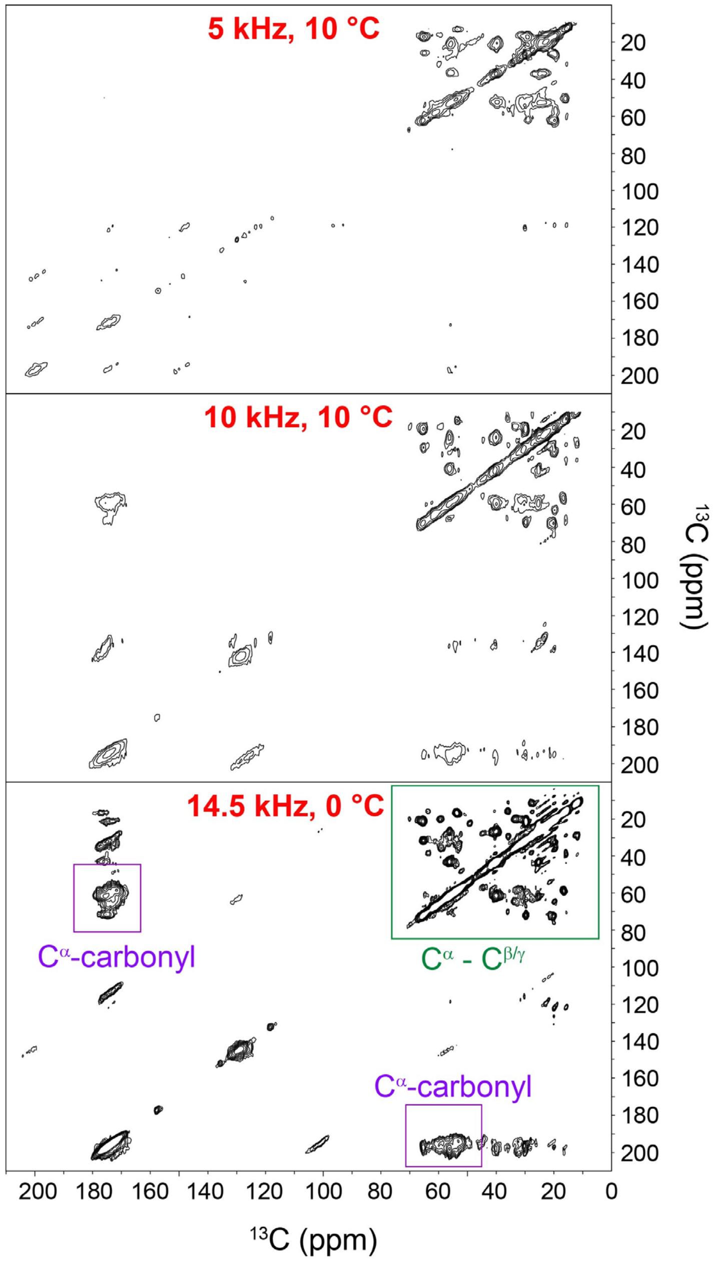

Figure S8. ^{13}C-^{13}C CP-DARR spectra of globomycin-bound $^{13}C/^{15}N$-labelled LspA in the LCP acquired at varying MAS frequencies and temperature settings. Spectra were collected using the 4-mm $^{1}H/^{13}C/^{15}N$ CPMAS probe with 750 μs CP and high-power ^{1}H decoupling. It is important to note that the DARR measurements at 10 and 14.5 kHz MAS (middle and lower panels) were inadvertently acquired with the incorrect MAS data collection settings. The MAS frequency parameter, that should be set equal to the MAS frequency employed in the measurement, was left at 5 kHz even though the MAS frequencies used in subsequent measurements had been increased. Therefore, the spectrum acquired at a MAS frequency of 5 kHz is calibrated properly while the two other spectra are not. As a result of this erroneous instrumental setting, the signal in the two bottom panels is stretched in the indirect

dimension (vertical axis) and the ^{13}C ppm values are not accurate. Nevertheless, the signal intensity, SNR, and resolution remain unaffected, and the panels may still be compared in terms of overall signal quality. We include the data in the two lower panels here primarily to demonstrate proof-of-concept and to show that DARR data can indeed be recorded with membrane protein-laden LCP samples and that an improvement in the quality of the signal can be achieved by adjusting experimental conditions.

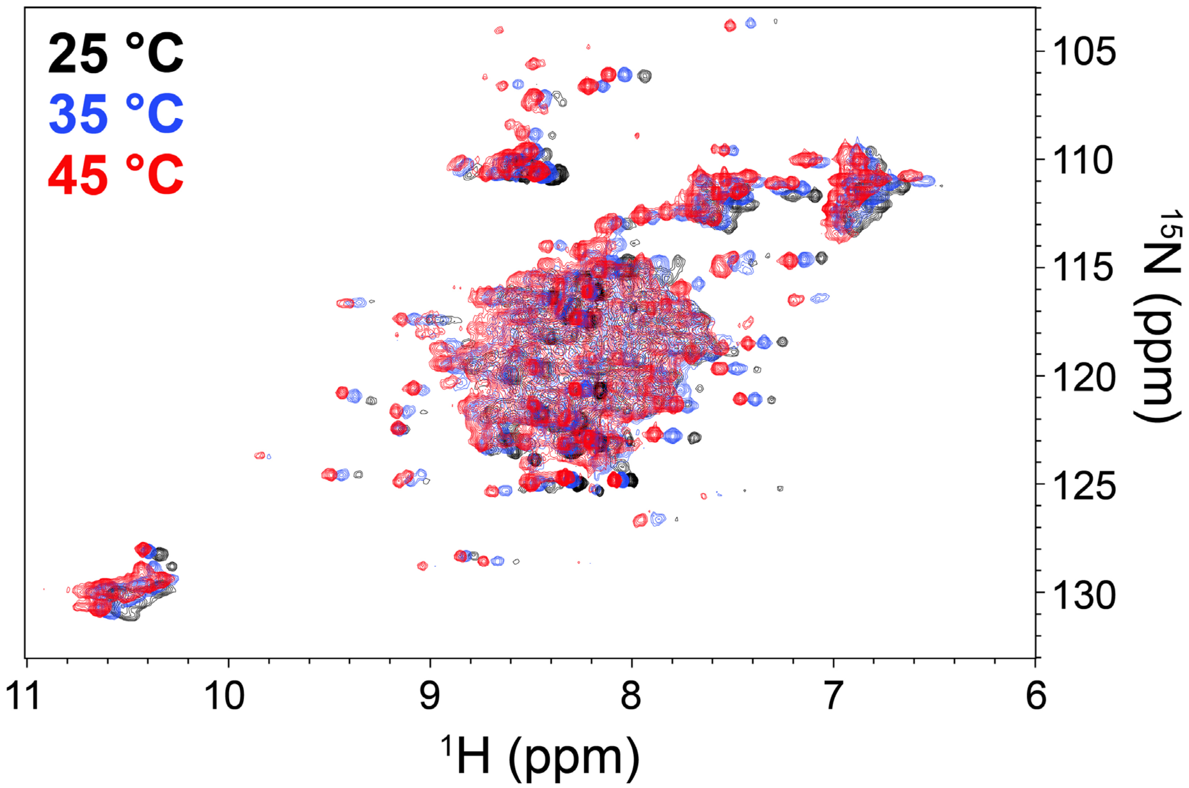

Figure S9. The effect of temperature on the ^{1}H-^{15}N HSQC spectrum of globomycin-bound ^{15}N-labelled LspA. Solution spectra were collected on samples containing 100 µM protein in 50 mM MES-NaOH pH 6.15, 150 mM NaCl, 0.14 %(w/v) FC-12, and 10 %(v/v) D_2O at 25 °C (black), 35 °C (blue), and 45 °C (red) using an 800 MHz NMR spectrometer equipped with a TCI CryoProbe.

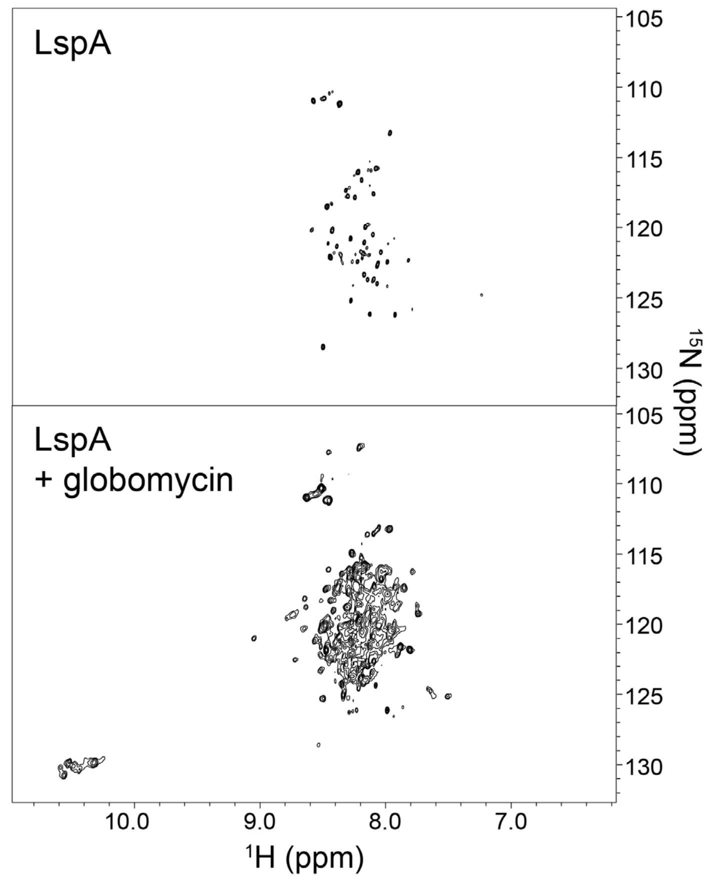

Figure S10. ^{1}H-^{15}N TROSY spectra of globomycin-free and globomycin-bound $^{2}H/^{13}C/^{15}N$-labelled LspA in FC-12 micelles recorded at 25 °C and 45 °C, respectively using an 800 MHz NMR spectrometer equipped with a TCI CryoProbe. Because the globomycin-free and globomycin-bound LspA spectra were recorded at different temperatures, a direct comparison between the two is not appropriate.

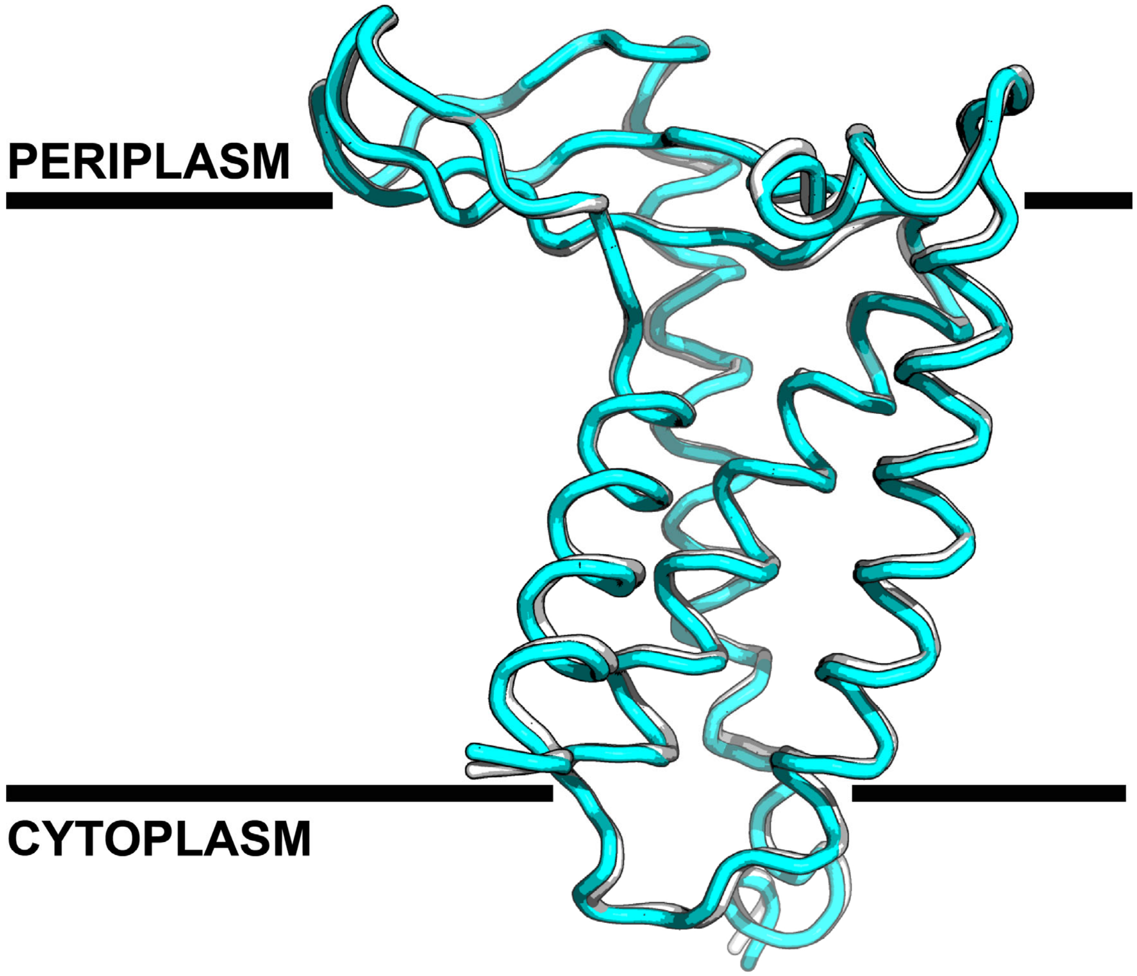

Figure S11. Alignment of *in meso* X-ray diffraction structures of unlabelled LspA (grey, PDB ID 5DIR) and $^{13}C/^{15}N$-labelled (cyan, PDB ID 9EMZ, this study) LspA in complex with globomycin. The structures of the labelled and unlabelled protein are virtually identical with a root-mean-square deviation of 0.36 Å for backbone C^{α} atoms. Approximate membrane boundaries are indicated by horizontal lines. Globomycin is omitted from the models for clarity.

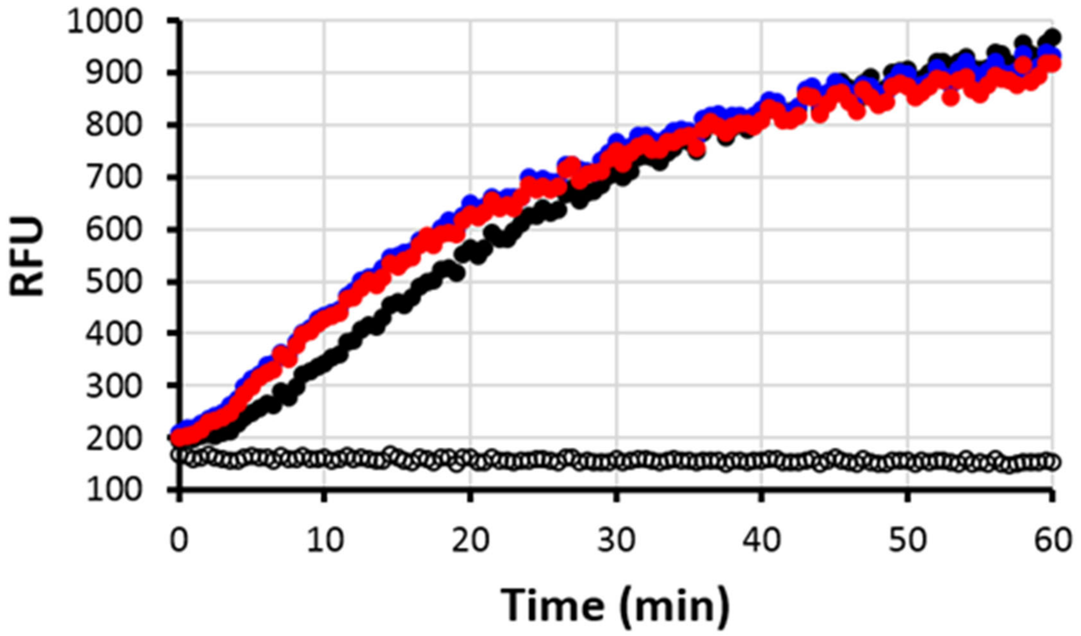

Figure S12. Progress curves for FRET activity assays used to monitor the endopeptidase activity of LspA with a FRET-labelled lipopeptide substrate (4). The activity of $^{13}C/^{15}N$-labelled LspA (duplicate assays shown as red and blue circles) was measured alongside unlabelled LspA (black full circles) and globomycin-treated $^{13}C/^{15}N$-labelled LspA (black open circles). The assay was conducted in samples containing 0.1 µM enzyme, 100 mM MES-NaOH pH 5.6, 150 mM NaCl, 80 µM FRET lipopeptide substrate, and 0.05 %(w/v) lauryl maltose neopentyl glycol (LMNG) in a reaction volume of 50 µL.

Supplemental Tables

Table S1. Parameters employed in the acquisition and processing of 1D ^{1}H MAS ssNMR spectra for gramicidin-LCP samples.

**Figure**	2A
**MAS frequency (kHz)**	5
**Temperature (°C)**	20
**Carrier frequency (MHz)**	800.1809527
**Spectral width (ppm)**	20.0275
**Acquisition time (s)**	0.5111808
**Number of scans**	8
**Size of FID (TD)**	8,192
**Size of processed spectrum (SI)**	16,384
**Recycle delay (D1), (s)**	3
**Window function (WDW)**	Exponential (EM)
**Line broadening (LB), (Hz)**	1.0

Table S2. Parameters employed in the acquisition and processing of direct excitation 1D ^{13}C MAS ssNMR spectra for gramicidin-LCP samples.

**Figure**	S5
**MAS frequency (kHz)**	5
**Temperature (°C)**	20
**Carrier frequency (MHz)**	201.2278720
**Spectral width (ppm)**	298.4679
**Acquisition time (s)**	0.1363968
**Number of scans**	8
**Size of FID (TD)**	8,192
**Size of processed spectrum (SI)**	16,384
**Recycle delay (D1), (s)**	3
**Window function (WDW)**	Gaussian (GM)
**Line broadening (LB), (Hz)**	-10
**Gaussian max. position**	0.01

Table S3. Parameters employed in the acquisition and processing of 1D ^{1}H-^{13}C CP-MAS ssNMR spectra for gramicidin-LCP samples.

**Figure**	2B
**MAS frequency (kHz)**	7.5
**Temperature (°C)**	0, 10, and 20
**Carrier frequency (MHz)**	800.1766330 (^{1}H), 201.2290210 (^{13}C)
**Spectral width (ppm)**	496.9462
**Acquisition time (s)**	0.0102400
**Number of scans**	512
**Size of FID (TD)**	1,024
**Size of processed spectrum (SI)**	2,048
**Recycle delay (D1), (s)**	3
**CP contact time (^{1}H-^{13}C) (µs)**	750
**Gradient**	100%–80%
**^{1}H Ramp shape**	linear
**^{1}H Ramp carrier frequency (MHz)**	800.1790325
**^{1}H Ramp power (kHz)**	70
**90° ^{1}H CP spinlock pulse power (kHz)**	50
**90° ^{1}H hard pulse power (kHz)**	25
**Acquisition decoupling type**	^{1}H, SPINAL-64 (5)
**Acquisition decoupling power (kHz)**	70
**Window function (WDW)**	Gaussian (GM)
**Line broadening (LB), (Hz)**	30
**Gaussian max. position**	-10

Table S4. Parameters employed in the acquisition and processing of direct excitation 1D ^{13}C MAS ssNMR spectra for LspA-globomycin-LCP samples.

**Figure**	S6
**MAS frequency (kHz)**	5
**Temperature (°C)**	20
**Carrier frequency (MHz)**	201.2278720
**Spectral width (ppm)**	298.4679
**Acquisition time (s)**	0.1363968
**Number of scans**	8
**Size of FID (TD)**	4,096
**Size of processed spectrum (SI)**	16,384
**Recycle delay (D1), (s)**	3
**Window function (WDW)**	SINE
**Sine bell shift (SSB)**	0

Table S5. Parameters employed in the acquisition and processing of 1D ^{1}H-^{15}N CP-MAS ssNMR spectra for LspA-globomycin-LCP samples.

**Figure**	3A
**MAS frequency (kHz)**	10
**Temperature (°C)**	10
**Carrier frequency (MHz)**	800.1766330 (^{1}H), 81.0845100 (^{15}N)
**Spectral width (ppm)**	740.7094
**Acquisition time (s)**	0.0170496
**Number of scans**	1,024
**Size of FID (TD)**	2,048
**Size of processed spectrum (SI)**	16,384
**Recycle delay (D1), (s)**	1
**CP contact time (^{1}H-^{15}N) (µs)**	750
**Gradient**	100%–80%
**^{1}H Ramp shape**	linear
**^{1}H Ramp carrier frequency (MHz)**	800.1790325
**^{1}H Ramp power (kHz)**	70
**90° ^{1}H CP spinlock pulse power (kHz)**	50
**90° ^{1}H hard pulse power (kHz)**	25
**Acquisition decoupling type**	^{1}H, SPINAL-64 (5)
**Acquisition decoupling power (kHz)**	70
**Window function (WDW)**	SINE
**Sine bell shift (SSB)**	0

Table S6. Parameters employed in the acquisition and processing of 1D ^{1}H-^{13}C CP-MAS ssNMR spectra for LspA-globomycin-LCP samples.

**Figure**	3B
**MAS frequency (kHz)**	5 and 14.5
**Temperature (°C)**	0 and 10
**Carrier frequency (MHz)**	800.1766330 (^{1}H), 201.2290210 (^{13}C)
**Spectral width (ppm)**	496.9462
**Acquisition time (s)**	0.0102400
**Number of scans**	512
**Size of FID (TD)**	2,048
**Size of processed spectrum (SI)**	16,384
**Recycle delay (D1), (s)**	3
**CP contact time (^{1}H-^{13}C) (µs)**	750
**Gradient**	100%–80%
**^{1}H Ramp shape**	linear
**^{1}H Ramp carrier frequency (MHz)**	800.1790325
**^{1}H Ramp power (kHz)**	70
**90° ^{1}H CP spinlock pulse power (kHz)**	50
**90° ^{1}H hard pulse power (kHz)**	25
**Acquisition decoupling type**	^{1}H, SPINAL-64 (5)
**Acquisition decoupling power (kHz)**	70
**Window function (WDW)**	SINE
**Sine bell shift (SSB)**	0

Table S7. Parameters employed in the acquisition and processing of 2D ^{15}N-detected ^{1}H saturation transfer difference ssNMR spectra for LspA-globomycin-LCP samples.

**Figure**	4
**MAS frequency (kHz)**	14.5
**Temperature (°C)**	10
**Carrier frequency (MHz)**	800.1766330 (^{1}H); 81.0845100 (^{15}N)
**Spectral width (ppm)**	5.1605 (^{1}H); 598.6802 (^{15}N)
**Acquisition time (s)**	0.0154988 (^{1}H); 0.0105472 (^{15}N)
**Number of scans**	512
**Size of FID (TD)**	128 (^{1}H); 1,024 (^{15}N)
**Size of processed spectrum (SI)**	1,024 (^{1}H); 2,048 (^{15}N)
**Recycle delay (D1), (s)**	2
**Mixing time (ms)**	400
**CP contact time (^{1}H-^{15}N) (µs)**	750
**Gradient**	100%–80%
**^{1}H Ramp shape**	Gaussian
**^{1}H Ramp carrier frequency (MHz)**	800.1790325
**^{1}H Ramp power (kHz)**	70
**90° ^{1}H CP spinlock pulse power (kHz)**	50
**90° ^{1}H hard pulse power (kHz)**	25
**Evolution decoupling type**	^{1}H; SPINAL-64 (5)
**Evolution decoupling power (kHz)**	70
**Evolution decoupling carrier frequency (MHz)**	800.1790325
**Acquisition decoupling type**	^{1}H; SPINAL-64 (5)
**Acquisition decoupling power (kHz)**	70
**Window function (WDW)**	Gaussian (GM)
**Line broadening (LB), (Hz)**	-1.0 (^{1}H); -1.0 (^{15}N)
**Gaussian max. position**	0.001 (^{1}H); 0.1 (^{15}N)

Table S8. Parameters employed in the acquisition and processing of 2D ^{15}N-$^{13}C^{\alpha}$ (NCA) SPECIFIC CP ssNMR spectra for LspA-globomycin-LCP samples.

**Figure**	S7
**MAS frequency (kHz)**	14.5
**Temperature (°C)**	0
**Carrier frequency (MHz)**	201.2259960 (^{13}C); 81.0845100 (^{15}N)
**Spectral width (ppm)**	219.89 (^{13}C); 740.00 (^{15}N)
**Acquisition time (s)**	0.0231424 (^{13}C); 0.0010666 (^{15}N)
**Number of scans**	1,024
**Size of FID (TD)**	1,024 (^{13}C); 64 (^{15}N)
**Size of processed spectrum (SI)**	2,048 (^{13}C); 128 (^{15}N)
**Recycle delay (D1), (s)**	1.5
**Indirect dimension**	^{15}N
**CP contact time (^{1}H-^{15}N) (µs)**	750
**CP Mixing time (ms)**	6
**Gradient**	100%–80%
**^{1}H Ramp shape**	Gaussian
**^{1}H Ramp carrier frequency (MHz)**	800.1790325
**^{1}H Ramp power (kHz)**	70
**90° ^{1}H CP spinlock pulse power (kHz)**	50
**90° ^{1}H hard pulse power (kHz)**	25
**Heteronuclear decoupling field strength (kHz)**	50
**Evolution decoupling type**	^{1}H; SPINAL-64 (5)
**Evolution decoupling power (kHz)**	70
**Evolution decoupling carrier frequency (MHz)**	800.1790325
**Acquisition decoupling type**	^{1}H; SPINAL-64 (5)
**Acquisition decoupling power (kHz)**	70
**^{15}N carrier frequency (ppm)**	119
**^{13}C carrier frequency (ppm)**	53
**Window function (WDW)**	Gaussian (GM)
**Line broadening (LB), (Hz)**	-1.0 (^{13}C); -1.0 (^{15}N)
**Gaussian max. position**	0.002 (^{13}C); 0.001 (^{15}N)

Table S9. Parameters employed in the acquisition and processing of 2D ^{13}C-^{13}C-DARR ssNMR spectra for LspA-globomycin-LCP samples.

**Figure**	S8
**MAS frequency[a] (kHz)**	5, 10, and 14.5
**Temperature (°C)**	0, 10, and 20
**Carrier frequency (MHz)**	201.2290210
**Spectral width (ppm)**	219.8877
**Acquisition time (s)**	0.0202752 (direct ^{13}C); 0.0012722 (indirect ^{13}C)
**Number of scans**	1,024
**Size of FID (TD)**	2,048; 128
**Size of processed spectrum (SI)**	2,048; 2,048
**Recycle delay (D1), (s)**	3
**Mixing time (ms)**	400
**CP contact time (^{1}H-^{13}C) (µs)**	750
**Gradient**	100%–80%
**^{1}H Ramp shape**	linear
**^{1}H Ramp carrier frequency (MHz)**	800.1790325
**^{1}H Ramp power (kHz)**	70
**90° ^{1}H CP spinlock pulse power (kHz)**	50
**90° ^{1}H hard pulse power (kHz)**	25
**^{13}C π/2 field strength (kHz)**	50
**Evolution decoupling type**	^{1}H; SPINAL-64 (5)
**Evolution decoupling power (kHz)**	70
**Evolution decoupling carrier frequency (MHz)**	800.1790325
**Acquisition decoupling type**	^{1}H; SPINAL-64 (5)
**Acquisition decoupling power (kHz)**	70
**Window function (WDW)**	Gaussian (GM)
**Line broadening (LB), (Hz)**	-1.0 (^{13}C); -1.0 (^{13}C)
**Gaussian max. position**	0.002 (^{13}C); 0.002 (^{13}C)

[a] Note that the MAS frequency parameter for the 2D ^{13}C-^{13}C-DARR experiments conducted at MAS frequencies of 10 and 14.5 kHz was inadvertently kept at 5 kHz during data acquisition. See legend to **Figure S8** and the main text for more details.

Table S10. Parameters employed in the acquisition and processing of 2D ^{1}H-^{15}N-HSQC solution NMR for unbound and globomycin-bound LspA samples.

**Figure**	5 and S9
**Temperature (°C)**	25, 35, and 45
**Carrier frequency (MHz)**	800.1766330 (^{1}H); 81.0845100 (^{15}N)
**Spectral width (ppm)**	16.0186 (^{1}H); 40.0000 (^{15}N)
**Acquisition time (s)**	0.07987 (^{1}H); 0.03945 (^{15}N)
**Number of scans**	96
**Size of FID (TD)**	1,024 (^{1}H); 128 (^{15}N)
**Size of processed spectrum (SI)**	2,048 (^{1}H); 256 (^{15}N)
**Recycle delay (D1), (s)**	3
**Window function (WDW)**	QSINE
**Sine bell shift (SSB)**	2 (^{1}H); 2 (^{15}N)

Table S11. Parameters employed in the acquisition and processing of 2D ^{1}H-^{15}N-TROSY solution NMR for unbound and globomycin-bound LspA samples.

**Figure**	S10
**Temperature (°C)**	25 and 45
**Carrier frequency (MHz)**	800.1766330 (^{1}H); 81.0845100 (^{15}N)
**Spectral width (ppm)**	16.0186 (^{1}H); 40.0000 (^{15}N)
**Acquisition time (s)**	0.07987 (^{1}H); 0.03945 (^{15}N)
**Number of scans**	128
**Size of FID (TD)**	1,024 (^{1}H); 128 (^{15}N)
**Size of processed spectrum (SI)**	2,048 (^{1}H); 256 (^{15}N)
**Recycle delay (D1), (s)**	3
**Window function (WDW)**	QSINE
**Sine bell shift (SSB)**	2 (^{1}H); 2 (^{15}N)

Table S12. X-ray data collection, processing, and refinement statistics for the *in meso* structure of $^{13}C/^{15}N$-labelled LspA in complex with globomycin

Data Collection	
**Light source**	SLS, PXII
**Wavelength (Å)**	1.00003
**Space group**	*C*2
**Cell constants (Å)**	113.991, 106.086, 85.919
**ß (°)**	97.54
**Resolution^a (Å)**	85.18-3.00 (3.31-3.00)
**Reflections**	23,890 (1,369)
**Unique reflections**	10,425 (522)
**Multiplicity**	2.3 (2.6)
**I/σ(I)**	4.3 (1.5)
**Completeness (%)**	72.7 (67.3)
**R_{meas}^b (%)**	0.155 (0.759)
**R_{pim}^c (%)**	0.096 (0.454)
**$CC_{1/2}$**	0.99 (0.57)
Refinement	
**R_{work}**	24.8
**R_{free}**	30.3
**rmsd bonds (Å)**	0.003
**rmsd angles (°)**	0.605
Number of molecules in the asymmetric unit	
**Protein chains**	4
**Globomycin**	4
**Water**	12
**Average B-factor (Å^2)**	55.11
**Clashscore**	8.18
Ramachandran analysis^d, % residues in	
**Favoured regions**	95.62
**Allowed regions**	4.38
**PDB code**	9EMZ

[a]Values in parentheses correspond to the highest resolution shell

[b]$R_{meas} = \sum_{hkl} \sqrt{n/n-1} \sum_l |I_i(hkl) - \langle I(hkl) \rangle| / \sum_{hkl} \sum_i I_i(hkl)$

[c]$R_{pim} = \sum_{hkl} \sqrt{1/n-1} \sum^{n}_{i=1} |I_i(hkl) - \langle I(hkl) \rangle| / \sum_{hkl} \sum_i I_i(hkl)$

[d]Calculated in MolProbity

Table S13. $^{1}H^{N}$ Linewidths[a] from the ^{1}H-^{15}N TROSY spectrum of globomycin-free $^{2}H/^{13}C/^{15}N$-labelled LspA in FC-12 micelles. The spectrum was collected with 50 μM LspA at 25 °C (**Fig. S10**, top panel).

**^{1}H δ (ppm)**	**^{15}N δ (ppm)**	**$^{1}H^{N}$ linewidth (Hz)**	**^{1}H δ (ppm)**	**^{15}N δ (ppm)**	**$^{1}H^{N}$ linewidth (Hz)**
8.4	128.7	16.9	8.2	120.8	13.6
7.8	126.4	12.1	8.0	120.5	15.7
8.0	126.3	14.0	8.5	120.2	24.7
8.2	125.3	12.9	8.4	120.2	19.5
8.0	124.1	14.1	8.1	120.0	16.2
8.1	123.8	15.0	8.4	118.5	19.1
8.0	123.8	16.5	8.4	118.3	19.6
8.1	123.4	14.4	8.2	117.8	15.0
8.0	122.8	15.7	8.2	117.7	18.9
8.0	122.7	15.0	8.0	117.5	16.4
8.1	122.5	15.8	8.2	117.3	17.0
7.9	122.5	13.9	8.2	117.1	23.0
7.7	122.4	15.7	8.1	116.5	13.6
8.1	122.2	15.9	8.1	115.9	16.1
8.4	122.2	16.3	8.0	115.8	35.7
8.3	122.1	17.4	8.0	115.7	22.8
8.1	121.9	31.2	7.9	113.1	14.5
8.0	121.8	14.8	8.3	111.0	18.9
8.1	121.5	17.1	8.5	110.7	16.7
8.3	121.4	14.2	8.4	110.6	29.3
8.4	121.2	14.3	8.4	110.2	15.5
8.1	121.1	14.6			
**Average linewidth (Hz)**			17.4 ± 5		

[a] Linewidths of resolved cross-peaks (43 out of a total of 44) were measured in CCPN (6). Resonances with linewidths ≥ 40 Hz were considered outliers and were excluded from the analysis.

Table S14. $^1H^N$ Linewidths[a] from the ^1H-^{15}N TROSY spectrum of globomycin-bound $^2H/^{13}C/^{15}N$-labelled LspA in FC-12 micelles. The spectrum was collected with 50 μM LspA at 45 °C (**Fig. S10**, bottom panel).

^{1}H δ (ppm)	^{15}N δ (ppm)	$^1H^N$ linewidth (Hz)	^{1}H δ (ppm)	^{15}N δ (ppm)	$^1H^N$ linewidth (Hz)
8.5	128.1	23.9	6.8	112.4	37.4
8.0	122.1	22.1	8.1	117.0	27.3
8.4	121.6	24.8	8.1	116.3	30.9
8.2	115.4	24.1	8.2	115.9	20.0
8.1	120.5	23.1	7.9	112.5	19.2
8.3	120.3	22.1	8.4	110.4	25.2
8.1	120.0	24.5	8.6	110.2	23.4
8.0	121.2	25.8	8.3	121.5	35.2
8.4	121.6	24.8	8.4	120.8	26.7
**Average linewidth (Hz)**			25.6 ± 5		

[a] Linewidths of resolved cross-peaks (18 out of a total of 32) were measured in CCPN (6). Resonances with linewidths ≥ 40 Hz were considered outliers and were excluded from the analysis.

Supplemental Calculations

The question

What concentration of peptide in aqueous solution is needed to prepare 50 μL of peptide-reconstituted LCP by the standard coupled syringe mixing method (1) to achieve a lipid-to-peptide molar ratio of 20:1?

Given

Molecular weight of monoolein, 356.5 g × mol^{-1}

Molecular weight of gramicidin, 1,880 g × mol^{-1}

50 μL of mesophase is prepared by the coupled syringe method typically by combining 30 μL of monoolein with 20 μL of aqueous solution.

Assumptions

- The density of monoolein is 1 g × mL^{-1}. Accordingly, volume (as in μL) and mass (as in mg) can be used interchangeably.
- Upon mixing and mesophase formation, the volumes of monoolein and peptide solution are additive.

The solution

To make 50 μL of gramicidin-laden mesophase, one starts with 30 μL (30 mg) monoolein which corresponds to (0.03 g monoolein) / (356.5 g × mol^{-1}) = 8.42 × 10^{-5} mol monoolein.

A monoolein/gramicidin molar ratio of 20:1 corresponds to (8.42 × 10^{-5} mol/20) = 4.2 × 10^{-6} mol gramicidin and to (4.2 × 10^{-6} mol × 1,880 g × mol^{-1} × 10^3 mg × g^{-1}) = 7.9 mg gramicidin.

To form the peptide-laden LCP, 30 μL of monoolein is combined with 20 μL of aqueous solution (a 3/2 volume or mass ratio) via the coupled syringe mixing method.

Therefore, the 7.9 mg of gramicidin must be "contained" in the 20 μL of aqueous solution. This corresponds to a gramicidin concentration of (7.9 mg gramicidin / 20 μL aqueous solution = 0.395 mg × μL^{-1}) = **395 mg × mL^{-1}.** After forming the LCP by combining the 20 μL of peptide solution with 30 μL of molten monoolein (a 3/2 volume or mass ratio) the final concentration of gramicidin in the mesophase overall is (395 mg × mL^{-1} × 2/5) = **158 mg × mL^{-1}**.

Calculation S1. To determine the concentration of peptide in aqueous solution that is needed to prepare 50 μL of peptide-reconstituted LCP by the standard coupled syringe mixing method to achieve a lipid-to-peptide molar ratio of 20:1.

Supplemental References

1. Caffrey, M., and Cherezov, V. 2009. Crystallizing membrane proteins using lipidic mesophases. *Nat. Protoc.* 4:706–731, doi: 10.1038/nprot.2009.31
2. Caboi, F., Borné, J., Nylander, T., Khan, A., Svendsen, A., and Patkar, S. 2002. Lipase action on a monoolein/sodium oleate aqueous cubic liquid crystalline phase—a NMR and X-ray diffraction study. *Colloids Surf. B* 26:159–171, doi: 10.1016/S0927-7765(02)00035-8
3. Baldus, M., Petkova, A. T., Herzfeld, J., and Griffin, R. G. 1998. Cross polarization in the tilted frame: assignment and spectral simplification in heteronuclear spin systems. *Mol. Phys.* 95:1197–1207, doi: 10.1080/00268979809483251
4. Olatunji, S., Yu, X., Bailey, J., Huang, C. Y., Zapotoczna, M., Bowen, K., Remškar, M., Müller, R., Scanlan, E. M., Geoghegan, J. A., et al. 2020. Structures of lipoprotein signal peptidase II from *Staphylococcus aureus* complexed with antibiotics globomycin and myxovirescin. *Nat. Commun.* 11:1–11, doi: 10.1038/s41467-019-13724-y
5. Fung, B.M., Khitrin, A.K., and Ermolaev, K. 2000. An improved broadband decoupling sequence for liquid crystals and solids. *J. Magn. Reson.* 142:97-101, doi: 10.1006/jmre.1999.1896
6. Vranken, W. F., Boucher, W., Stevens, T. J., Fogh, R. H., Pajon, A., Llinas, M., Ulrich, E. L., Markley, J. L., Ionides, J., and Laue, E. D. 2005. The CCPN data model for NMR spectroscopy: development of a software pipeline. *Proteins* 59:687–696, doi: 10.1002/prot.20449
